# Incremental Information from Repeated Loneliness Assessments in Middle-Aged and Older Adults

**DOI:** 10.3390/healthcare14183058

**Published:** 2026-09-17

**Authors:** Zhengqi Qiu, Yunzhi Huang, Juntao Kan, Lin Xu

**Affiliations:** 1 School of Public Health, Sun Yat-sen University, No. 74, 2nd Zhongshan Road, Guangzhou 510080, China; 2024390282@gzhmu.edu.cn; 2 The Institute of Mental Psychology, School of Health Management, Guangzhou Medical University, Guangzhou 510370, China; 3 Nutrilite Health Institute, Amway R&D Center, 720 Cailun Road, Shanghai 201203, China; emma.huang@amway.com

**Keywords:** loneliness trajectories, repeated assessment, adults, sex differences, depressive symptoms, physical function, cognition, longitudinal cohort

## Abstract

**Highlights:**

**What are the main findings?**

- Adding two later loneliness assessments produced statistically detectable but small out-of-sample information gains in women and men in CHARLS, with a more variable comparison in HRS.
- Associations were concentrated in depressive symptoms and everyday function, with smaller associations for cognition and little evidence of associations with metabolic or inflammatory markers.

**What are the implications of the main findings?**

- Repeated assessment can modestly improve the description of health burden in women and men, although its clinical utility remains unestablished.
- Functional and psychological outcomes merit priority in the future prospective validation of loneliness monitoring in midlife and later life.

**Abstract:**

Background/Objectives: Loneliness can persist, resolve or develop over time. We examined whether repeated assessments add information about health beyond an initial assessment and which health domains account for that information. Methods: The primary analysis included 4904 women aged ≥45 years in the China Health and Retirement Longitudinal Study (CHARLS). Loneliness was assessed in 2011, 2013 and 2015. The outcome combined worsening depressive symptoms, physical function and cognition over 2011–2018, although some physical measurements ended in 2015. Thus, exposure and outcome periods overlapped. We compared models using baseline information alone with models adding the later assessments, testing both in held-out participants. Post hoc analyses included 4287 men. The Health and Retirement Study (HRS) provided a longitudinal comparison in women and additional analyses in men; UK Biobank provided a baseline comparison in women only. Results: Repeated assessments modestly increased explained variation in CHARLS (averaged difference in R-squared [ΔR^2^] = 0.0178, 95% confidence interval [CI] 0.0103–0.0254 in women; 0.0148, 95% CI 0.0067–0.0228 in men). Associations were strongest for depressive symptoms and everyday function, smaller for cognition, and near null for metabolic and inflammatory markers. There was no clear evidence of sex or age differences in the tested associations. HRS gains were modest in women and imprecise in men. UK Biobank used a different joint loneliness and low-confiding measure. Conclusions: Repeated loneliness assessments added modest information, chiefly about psychological and functional burden. Overlapping measurement periods and differences between cohorts limit causal interpretation and generalizability. Clinical usefulness remains unestablished.

## 1. Introduction

Loneliness is associated with mortality [1], chronic disease [2], depressive symptoms [3], functional decline [4], and cognitive outcomes [5]. It also co-occurs with reduced physical activity, poorer self-rated health, sleep disturbance, disability, and barriers to care [6]. These associations have prompted multidomain approaches to psychological, functional, and cognitive health [7], but their interpretation remains unsettled: loneliness may operate as an exposure, reflect social disconnection, or mark pre-existing health deterioration [8,9]. Understanding these relationships requires attention to both the timing of measurements and the information gained from repeated assessment.

Loneliness and social isolation are related but non-equivalent constructs [10]. Loneliness reflects perceived insufficiency in the quality or quantity of social relationships, whereas social isolation describes a more objective lack of contact, partnership, or participation [11]. Either can occur without the other [12]. We therefore treated loneliness as the exposure of interest and adjusted for a limited measure of objective social isolation, while recognizing that neither construct fully captures relationship quality or perceived support.

A further question is whether loneliness is associated with health broadly or with particular outcome domains. Prior studies have linked loneliness with depressive symptoms [3], functional decline [4], cognitive decline or dementia [5,13], and mortality [1]. Loneliness and related social-disconnection measures have also been associated with physical health [14], while outcome-wide analyses show that associations can differ materially across domains [15,16]. Domain-specific results help distinguish broad associations from those concentrated in mood or everyday function. We used a psychological-functional-cognitive composite to summarize burden without assuming a generalized aging process, reported its domains separately, and treated inflammatory and metabolic measures as exploratory outcomes. Separate clinical and functional incident endpoints were evaluated over 2015–2018.

Loneliness changes over time, so one baseline answer cannot distinguish a temporary episode from a persistent state or identify later onset and remission [17]. Later-life trajectory studies confirm this heterogeneity [18,19]. In the Survey of Health, Ageing and Retirement in Europe (SHARE), chronic and transient loneliness had different risk-factor profiles, and chronic loneliness was associated with depressive symptoms over two years [20]. Among middle-aged Australian women, sustained or increasing loneliness was associated with poorer mental health-related quality of life, whereas physical health associations differed across trajectories [21]. In England, chronic and fluctuating loneliness patterns were associated with subsequent functional impairment [22]. Prior China Health and Retirement Longitudinal Study (CHARLS) work also linked loneliness trajectories with cardiovascular outcomes [23], whereas baseline loneliness alone did not clearly predict disability in another analysis [24]. Baseline loneliness has also been associated with depressive-symptom severity over 12 years in England [25]. These findings support assessing changes in loneliness. A separate question is whether later assessments contribute information beyond baseline loneliness and health. Reviews of repeated predictors and incremental value recommend an explicit baseline comparison with held-out evaluation [26,27]. We therefore asked how much information the 2013 and 2015 assessments added to 2011 loneliness and baseline health and whether that information differed across outcome domains.

CHARLS follows Chinese adults aged ≥45 years, with repeated psychosocial, cognitive, functional, and health assessments [28]. The original primary analysis focused on women, to examine loneliness in the context of life-course social relationships and psychological health [18,29]. This focus is relevant to the higher burden of depressive symptoms reported among older women than men in CHARLS [30]. A stress-and-coping perspective also suggests that family relationships, social participation, and access to support may be associated with psychological health differently in women and men [31]. In a different social context, older women in England experienced greater mental-health deterioration than men during the COVID-19 pandemic [32]. Although that later disruption cannot explain the earlier CHARLS observations, it illustrates how social circumstances may influence sex differences in mental health. Together, these considerations support examining women separately without assuming greater intrinsic stress susceptibility or a stronger loneliness–health association. We subsequently included all eligible men in post hoc sensitivity analyses and tested sex interactions.

Our primary aim was to determine whether repeated loneliness assessments add information about health burden beyond baseline loneliness and health. We also examined which health domains contributed to that information. We compared models using the first assessment alone with models adding the later assessments, evaluating both in participants held out from model fitting. The Health and Retirement Study (HRS) was included to examine these patterns in a US longitudinal cohort, while UK Biobank provided a separate baseline comparison of related psychological and physical phenotypes in a UK setting [33,34]. These complementary comparisons helped assess the scope of the findings while keeping the non-equivalent measures and effects separate. Variation by sex and age was examined in the additional sensitivity analyses.

## 2. Materials and Methods

### 2.1. Study Design and Analytic Population

The primary CHARLS observation period was 2011–2018 (seven years), with loneliness measured from 2011 to 2015. The prespecified primary analysis included women aged ≥45 years with observed 2011 loneliness, complete loneliness classification in 2011, 2013, and 2015, an aligned baseline burden score, and an observed 2018 primary composite. This fixed sample comprised 4904 women. The same eligibility rules were applied to men in additional post hoc sensitivity analyses, adding 4287 men and yielding 9191 adults for sex comparisons. These analyses complemented rather than replaced the original primary analysis in women.

We retained the cohort’s midlife-and-older age range to address a common measurement question across this population: whether a loneliness history adds information beyond baseline status. This does not assume that loneliness has the same causes or social meaning at every age. The pooled models adjusted for age, and age-stratified estimates and an interaction test examined variation in the association. Cohort selection was recorded at each stage, and 2018-observation and baseline-response-weight sensitivities were evaluated separately.

The analytic samples, ages, survey years, and sex restrictions for all three cohorts are summarized in Supplementary Table S27. Supplementary Tables S28–S30 provide the corresponding exposure, outcome, covariate, and missing-data definitions. The cohort comparisons used women as the primary comparison samples; men were included in additional CHARLS and HRS analyses but not in the UK Biobank baseline analysis.

### 2.2. Loneliness Trajectories and Social Isolation

Loneliness was measured with the direct Center for Epidemiologic Studies Depression Scale (CES-D) item asking how often the respondent felt lonely [35]. Responses of 3 or 4 defined high loneliness and responses of 1 or 2 defined not-high loneliness in 2011, 2013, and 2015. Five patterns were specified: never high, remitted, incident, intermittent, and persistent. The continuous score was (2 × L2011 + 2 × L2013 + 3 × L2015)/2, where L = 1 for high loneliness. The 2:2:3 weights represent the lengths of the 2011–2013, 2013–2015, and 2015–2018 intervals; they assign the last observed status to each interval rather than measure loneliness continuously. Because this approach assumes within-interval persistence, we repeated the primary and domain analyses with equal weights of 1:1:1. Baseline social isolation (unpartnered status and no social participation; range 0–2) was an adjustment variable, not an alternative exposure.

The five patterns were assigned from the three binary indicators in chronological order: never high, 000; remitted, 100; incident, 001, 010, or 011; intermittent, 101 or 110; and persistent, 111. Thus, the incident category includes a single high assessment in 2013 followed by a not-high assessment in 2015. These are observed response patterns, not diagnoses of chronic loneliness.

### 2.3. Observation-Interval Burden Outcomes

The primary outcome summarized psychological, functional, and cognitive worsening across the observation period rather than health changes occurring entirely after loneliness assessment. Psychological worsening was 2018 minus 2011 CES-D-9, obtained by removing the loneliness item from CES-D-10. Cognitive worsening was 2011 minus 2018 global cognition, which combined memory, orientation, serial subtraction, and figure copying. The physical-function domain averaged seven available standardized changes. Activities of daily living (ADL) limitations, seven-item mobility difficulties, and lower-body mobility difficulties were measured from 2011 to 2018; grip strength, walking-test time, chair-stand time, and balance were measured from 2011 to 2015. The latter components therefore ended at the final loneliness assessment. Individual mobility items were not entered again as additional components. Supplementary Table S28 specifies item content, score ranges, direction, and timing.

Physical-function components were standardized and averaged when at least two were available. The primary composite was then the equal-weighted mean of available psychological, physical-function, and cognitive domain scores, requiring at least two domains. Component means and standard deviations came from the analysis-ready source dataset before final-sample restriction and were not recalculated within the 4904 women. The resulting average is expressed in standardized-score units; it was not rescaled to have a standard deviation of one. An aligned 2011 burden score averaged available CES-D-9, worse cognition, worse grip strength, and ADL limitation scores. The composite was an internally constructed summary, not a validated biological-aging measure. Inflammation and metabolic changes were analyzed separately as exploratory domains, and a broader composite was retained as a sensitivity analysis [15].

The overlapping design addressed whether a loneliness history described health burden over the same period more fully than the first assessment alone. It did not address whether information available in 2011 entirely predicted future disease. Repeated loneliness measurements can capture health deterioration that has already begun, including changes preceding the 2013 and 2015 responses. We retained this primary question and separately examined outcomes occurring after the final measured loneliness assessment, as described below.

In response to the request for transparent measurement reporting, we reviewed the operational definitions and supporting documentation for the secondary outcomes. The available documentation for the imported cardiovascular–kidney–metabolic (CKM) measures did not provide the full component rules required to describe their construction explicitly. We therefore excluded stage progression and the original stage-derived cardiovascular endpoint, and we removed stage contributions from every affected alternative composite, baseline adjustment score, and completeness count. These secondary analyses were refitted, with their multiple-testing corrections recalculated. This post hoc revision improved the transparency of secondary-outcome reporting. The primary sample, exposure, outcome, aligned baseline score, and cross-validation specification were unchanged. The separate self-reported heart-disease/stroke endpoint for 2015–2018 was retained. Supplementary Table S28 gives the revised component rules.

### 2.4. Covariates and Statistical Analyses

The analyses served distinct purposes. Model comparisons quantified the information gained from later loneliness assessments; domain-specific analyses identified which outcomes contributed to the overall association. Sensitivity analyses examined whether the findings depended on scoring choices, sample restrictions or adjustment. Analyses of remission and incident outcomes addressed secondary questions.

Primary linear models used heteroskedasticity-consistent type 3 (HC3) standard errors [36] and adjusted for baseline age, education, rural residence, body mass index, ever smoking, alcohol use during the previous year, a count of 12 doctor-diagnosed chronic conditions, social isolation, and aligned baseline burden. Trajectory contrasts used never-high as the reference. The association models retained the original analysis-ready covariate fills; among the 4904 women, 588 BMI values and one chronic-condition count were filled. Missingness handling and cohort-specific covariate coding are detailed in Supplementary Tables S28–S30. The original robustness analyses evaluated alternative composites, inverse-probability weights, covariate additions, age restrictions, exposure coding, and a 2015–2018 change outcome adjusted for 2015 burden. Additional post hoc sensitivity analyses included equal weighting, age groups (45–59, 60–74, and ≥75 years), men, and cumulative-exposure-by-sex interactions. The retained domain and unadjusted cumulative-exposure families each comprised 11 outcomes; the sensitivity family comprised 19 specifications, and each equal-weight comparison family comprised 11 outcomes. Related secondary tests used Benjamini–Hochberg false discovery rate (FDR) correction [37].

To test incremental information directly, we compared a fixed baseline-only model with an otherwise identical repeated-assessment model. In CHARLS, the latter added 2013 and 2015 loneliness to 2011 status; in HRS, it added waves 10 and 12 to wave 8 status. Participants were assigned to identical folds in ten repeats of five-fold cross-validation using fixed seeds 20260808–20260817. Predictor imputation and categorical encoding were fitted within training folds; the outcomes retained their source-dataset standardization. R-squared (R^2^), root mean squared error (RMSE), mean absolute error (MAE), and calibration were reported. Sex-stratified sensitivity analyses used the same model specifications and seed schedule, with paired folds for each model comparison. This internal-validation design evaluates added statistical information [26,27,38]. Because the outcomes partly overlap loneliness assessment, held-out performance does not establish prospective prediction from baseline or resolve temporal ordering.

Among women with high loneliness in 2011, we examined social and family correlates of membership in the remitted pattern and contrasted 2011–2018 burden between remitted and non-remitted groups, with adjustment for baseline burden, loneliness intensity, and CES-D-9 where appropriate. The correlate models used the broader CKM-free 2011 burden score, whereas the remission–outcome contrast used the aligned primary baseline score (Supplementary Table S28). These descriptive variables were not assumed to have the same meaning at all ages; anticipated child help or transfers may be less salient for employed adults in their late forties than for retired or very old adults. The analyses were not interpreted as effects of reducing loneliness.

As secondary temporally separated analyses, women free of each outcome in 2015 were followed for corresponding incident status in 2018. Eligibility was determined separately for each endpoint from the full trajectory-eligible cohort rather than conditioned on availability of the primary composite. Modified Poisson models with robust standard errors estimated relative risks [39]. Separate binomial-logit models with the same covariates supplied bounded standardized risks and risk differences. Fixed-seed participant bootstrap intervals were used for the cumulative exposure contrast; trajectory-standardized risks and differences used the robust delta method. The seven CHARLS cumulative-exposure tests formed one Benjamini–Hochberg family, and the 28 nonreference trajectory contrasts formed a separate family [37]. HRS incident ADL limitation was analyzed under the harmonized specification. These outcomes were secondary and were not used to redefine the primary endpoint.

### 2.5. Cross-Cohort Comparisons

The cohorts were included for distinct purposes. CHARLS provided the primary analysis in a Chinese setting. HRS provided a longitudinal comparison in the United States using a related survey infrastructure and overlapping health domains rather than an externally validated version of the CHARLS model [33,40]. The HRS cross-validation sample included 2080 women aged ≥50 years at wave 8 (mean 64.67 years, SD 8.32); the complete-case primary association model included 2025 women. An additional sensitivity analysis included 1372 men. Loneliness was measured with the Hughes Three-Item Loneliness Scale at waves 8, 10, and 12 (2006, 2010, and 2014) [41]. The follow-up composite in the final female sample comprised depressive-symptom, ADL, and cognitive worsening from wave 8 to wave 13 (2016); no grip-change component was available in this sample. The direct CES-D item in CHARLS and indirect three-item scale in HRS differ in wording, scoring, and recall frame. Comparisons therefore concern directions and domains of association, not interchangeable exposure units or equal effect sizes.

UK Biobank recruited adults aged 40–69 years in 2006–2010 and provided a baseline comparison using questionnaire and assessment-center measurements [34]. Our eligible dataset contained 228,807 women aged ≥45 years; 182,571 contributed to the complete-case baseline-composite model (mean age 57.65 years, SD 6.80). The exposure required both often feeling lonely and never or almost never being able to confide in someone. It therefore represents a joint loneliness and low-confiding indicator rather than the CHARLS single-item or the HRS scale. The baseline composite summarized depressive symptoms, grip weakness, and C-reactive protein; outcome-specific sample sizes differed. No male-specific UK Biobank analysis was conducted. Repeated loneliness measures were not available in the local analytic extract, so UK Biobank was not used to test trajectories, temporal ordering, or the gain from repeated assessment. Exact measures and cohort-specific adjustment sets are given in Supplementary Tables S27–S30.

### 2.6. Ethics Approval and Informed Consent

This secondary analysis used de-identified data from CHARLS, HRS, and UK Biobank. CHARLS was approved by the Biomedical Ethics Committee of Peking University (IRB00001052–11015 and IRB00001052–11014), HRS by the University of Michigan IRB, and UK Biobank by the North West Multi-centre Research Ethics Committee (11/NW/0382). All parent-cohort procedures were performed in accordance with relevant institutional and national guidelines and regulations. Participants in the original CHARLS, HRS, and UK Biobank studies provided informed consent according to each cohort’s procedures. No additional consent was required for this secondary analysis.

### 2.7. Use of Generative AI

OpenAI Codex (GPT-5; OpenAI, San Francisco, CA, USA; accessed August and September 2026) was used for code generation, analysis quality checks, figure and table preparation, manuscript organization, and language editing. The authors reviewed and verified all outputs and remain fully responsible for the analyses, interpretation, and content of the manuscript.

## 3. Results

### 3.1. Study Population and Baseline Characteristics

**Figure 1:**
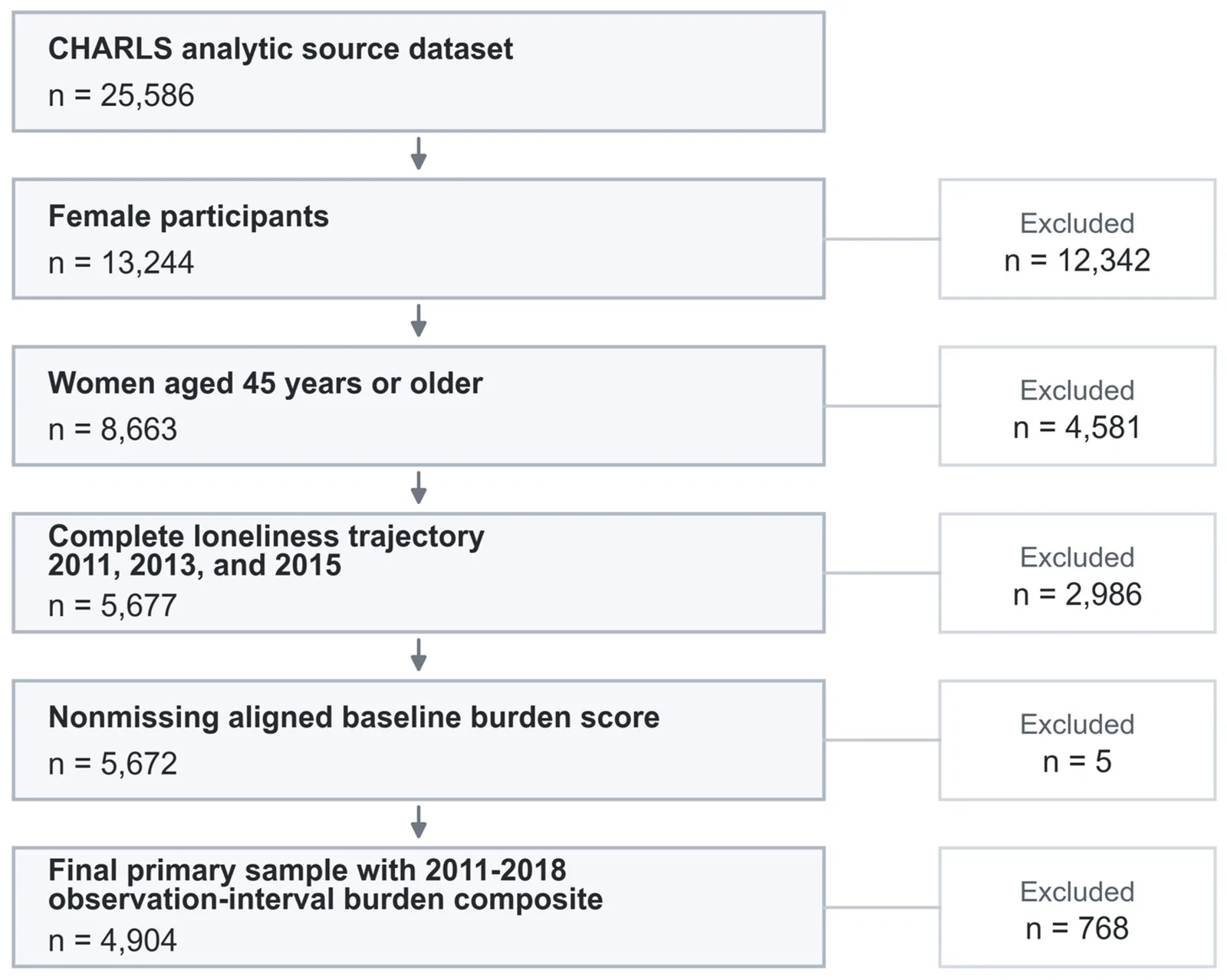
Study population flow. The diagram shows sequential selection from the CHARLS-derived analysis-ready source dataset to the fixed sample of 4904 women with complete loneliness classification, aligned baseline burden, and the primary 2011–2018 observation-interval composite.

The analysis-ready CHARLS-derived dataset contained 25,586 records. Sequential restrictions left 13,244 women: 8663 women aged ≥45 years, 7980 with 2011 loneliness observed, 5677 with complete 2011/2013/2015 loneliness classification, 5672 with an aligned baseline burden score, and 4904 with the primary composite (Figure 1; Supplementary Table S1). Applying the same final eligibility rules identified 4287 men; the sex-comparative sample therefore included 9191 adults. Of the primary sample of women, 74.4% contributed all three primary domains; most others contributed psychological and physical-function domains. Endpoint-specific secondary cohorts were constructed independently.

Baseline socioeconomic, social, and health profiles differed across patterns; the persistent group had greater baseline disadvantage than the never-high group, whereas the remitted group had a more favorable baseline profile than women whose loneliness did not remit (Supplementary Table S2). The primary coefficients are consequently conditional on measured baseline burden and covariates rather than marginal group differences.

### 3.2. Loneliness Trajectories and Observation-Interval Burden

**Figure 2:**
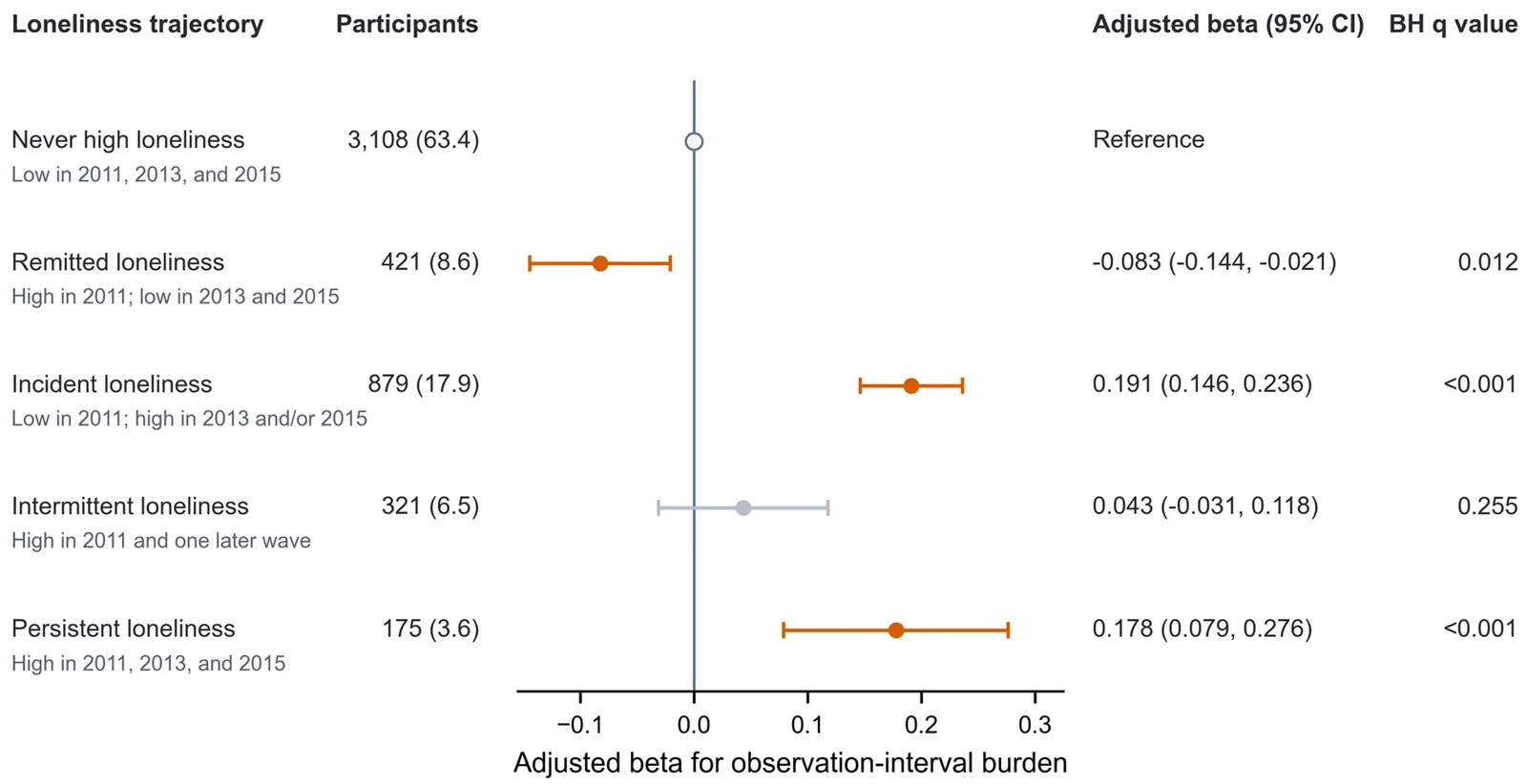
Loneliness trajectories and 2011–2018 observation-interval psychological-functional-cognitive burden among women in CHARLS aged ≥45 years. Points are adjusted beta coefficients and, horizontal bars are 95% confidence intervals; never-high is the reference. Models adjusted for age, education, rural residence, body mass index, smoking, alcohol use, chronic disease count, social isolation, and aligned baseline burden. The outcome interval partly overlaps trajectory ascertainment. Orange and grey denote Benjamini–Hochberg-adjusted q < 0.05 and q ≥ 0.05, respectively; the open circle denotes the reference group.

In the covariate- and baseline-burden-adjusted model, incident (β = 0.191, 95% confidence interval (CI) 0.146–0.236) and persistent high loneliness (β = 0.178, 95% CI 0.079–0.276) were associated with greater 2011–2018 observation-interval burden than never-high loneliness (Figure 2; Supplementary Table S3). The remitted group had lower adjusted burden (β = −0.083, 95% CI −0.144 to −0.021), whereas the intermittent group did not differ clearly (β = 0.043, 95% CI −0.031 to 0.118). Per two-year-scaled unit, assigned high-loneliness exposure was associated with a 0.066 standardized-score-unit greater burden (95% CI 0.047–0.085). The 2018-observation inverse-probability-of-censoring weighting (IPCW) estimate was similar (β = 0.066, 95% CI 0.047–0.085). These estimates describe conditional association across an overlapping observation interval, not a post-exposure effect.

### 3.3. Domain-Specific Outcomes

**Figure 3:**
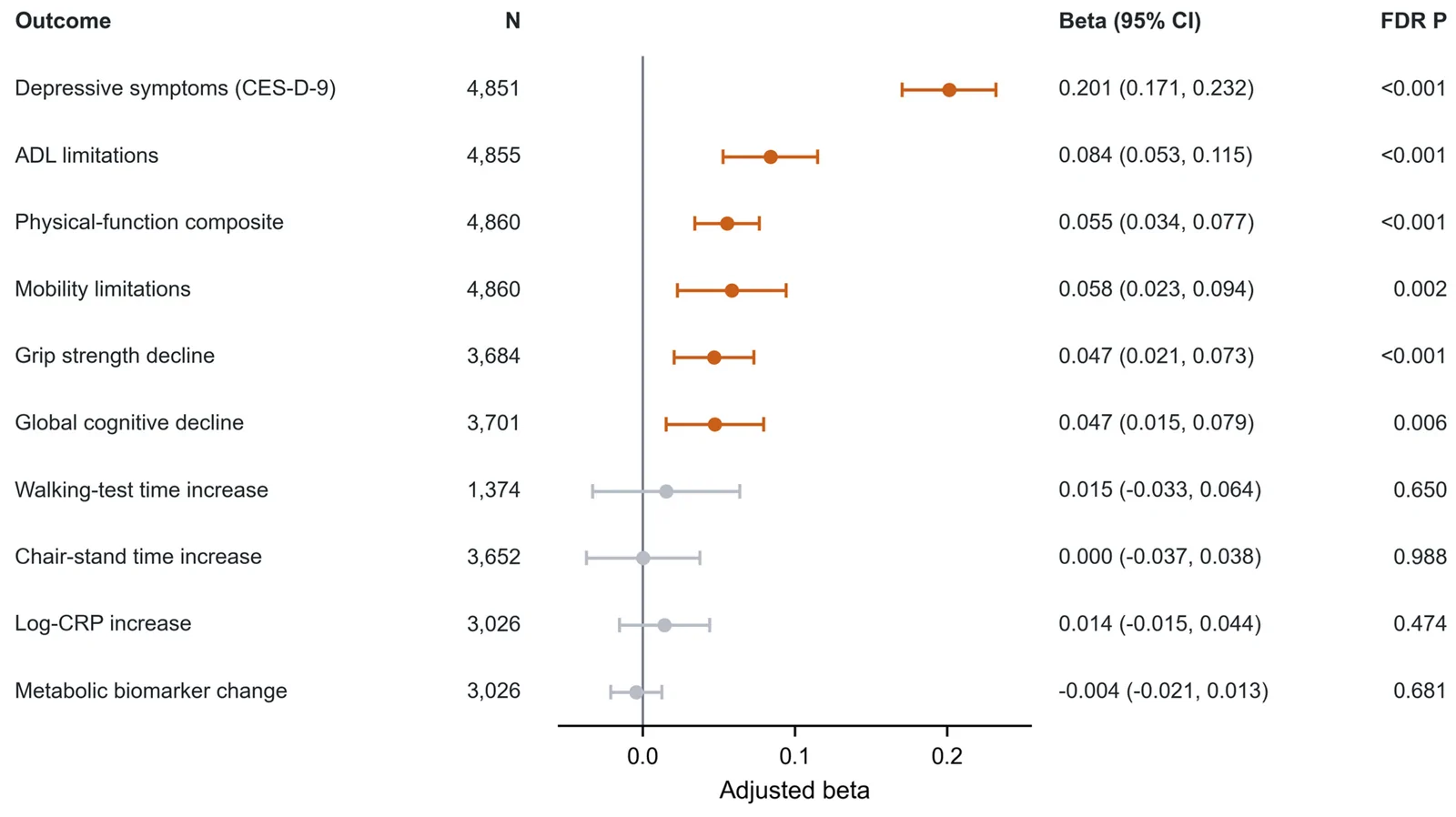
Domain-specific outcomes associated with two-year-scaled assigned high-loneliness exposure. One unit approximates two assigned, not continuously observed, high-loneliness years. Points show adjusted beta coefficients, and bars show 95% confidence intervals. CES-D-9 excludes the loneliness item; increased walking-test time indicates slowing. Biomarker outcomes are exploratory. Orange indicates FDR-adjusted *p* < 0.05, and grey indicates *p* ≥ 0.05 after correction across all 11 retained outcomes, including the primary composite reported in Table S4. Model-specific baseline adjustment is detailed in Table S28.

Domain decomposition showed the largest association for depressive-symptom worsening (β = 0.201, 95% CI 0.171–0.232), followed by ADL worsening (β = 0.084, 95% CI 0.053–0.115), the physical-function composite (β = 0.055, 95% CI 0.034–0.077), and cognitive decline (β = 0.047, 95% CI 0.015–0.079) per exposure unit (Figure 3; Supplementary Table S4). In contrast, metabolic deterioration was near null (β = −0.004, 95% CI −0.021–0.013), and inflammation was imprecise (β = 0.014, 95% CI −0.015–0.044). This pattern supports domain specificity rather than generalized biological aging.

### 3.4. Loneliness Remission and Social Context

**Figure 4:**
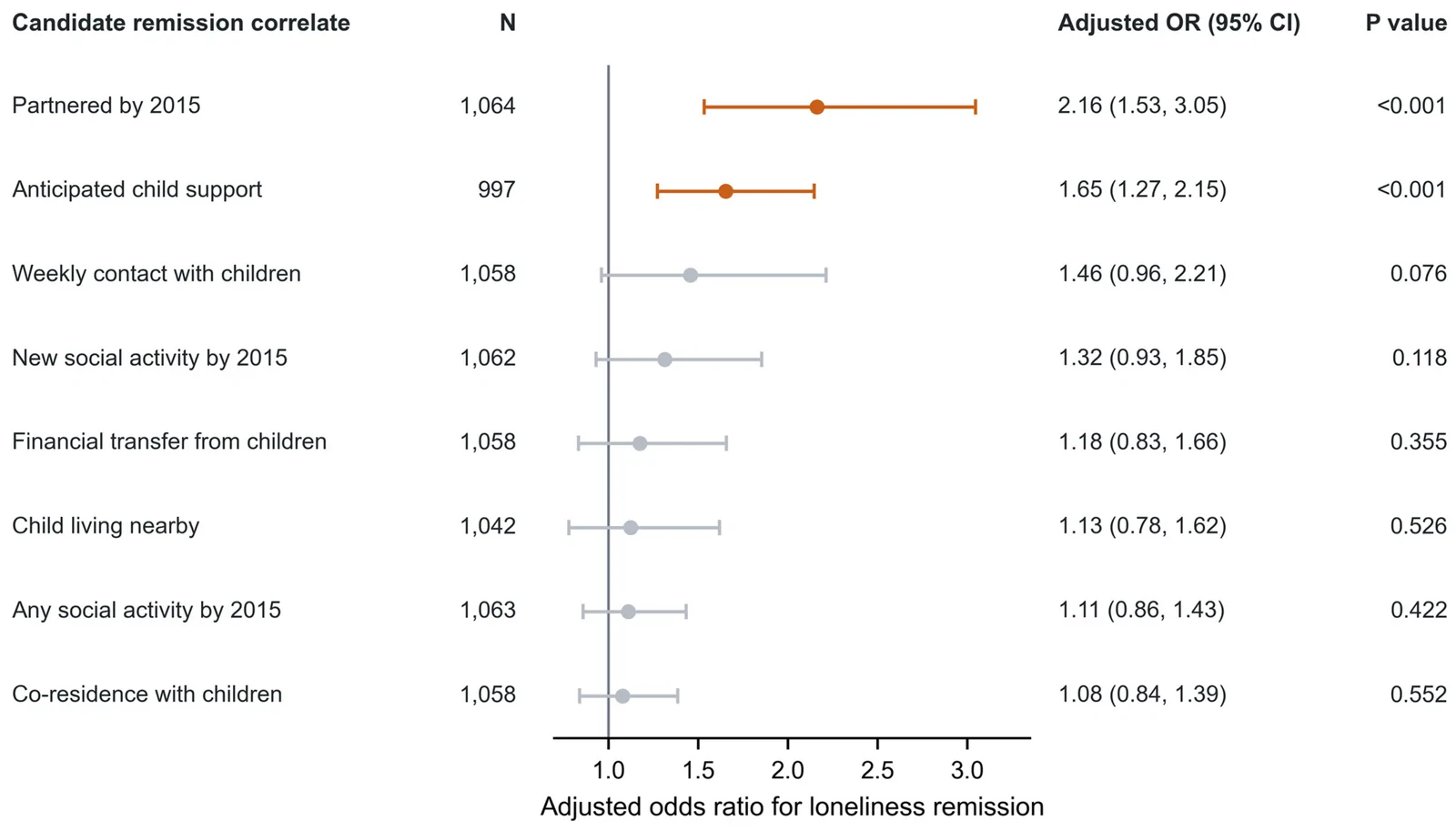
Correlates of membership in the remitted pattern among women with high loneliness in 2011. Remission was defined as high loneliness in 2011 followed by not-high loneliness in both 2013 and 2015. Points are adjusted odds ratios with 95% confidence intervals; these descriptive correlates are not intervention effects. Models adjusted for age, education, rural residence, BMI, chronic-condition count, social isolation, and broader CKM-free baseline burden. N denotes the complete-case sample for each correlate; *p* values are unadjusted across these exploratory models. Orange denotes *p* < 0.05 and grey denotes *p* ≥ 0.05.

Among women with high loneliness in 2011, partnership and anticipated help from children were associated with membership in the remitted pattern (Figure 4; Supplementary Table S7). Their relevance may depend on age and employment. These descriptive correlates were therefore not generalized to all adults aged ≥45 years or interpreted as intervention effects, and they were not used to select the primary model.

The remitted group entered follow-up with a more favorable measured profile than the non-remitted group (Supplementary Table S8). Although the adjusted contrast persisted in the observation-interval composite (Supplementary Table S9), residual baseline differences and overlapping exposure/outcome windows preclude a causal interpretation of the remission–burden contrast.

### 3.5. Sensitivity Analyses and Cross-Cohort Comparisons

**Figure 5:**
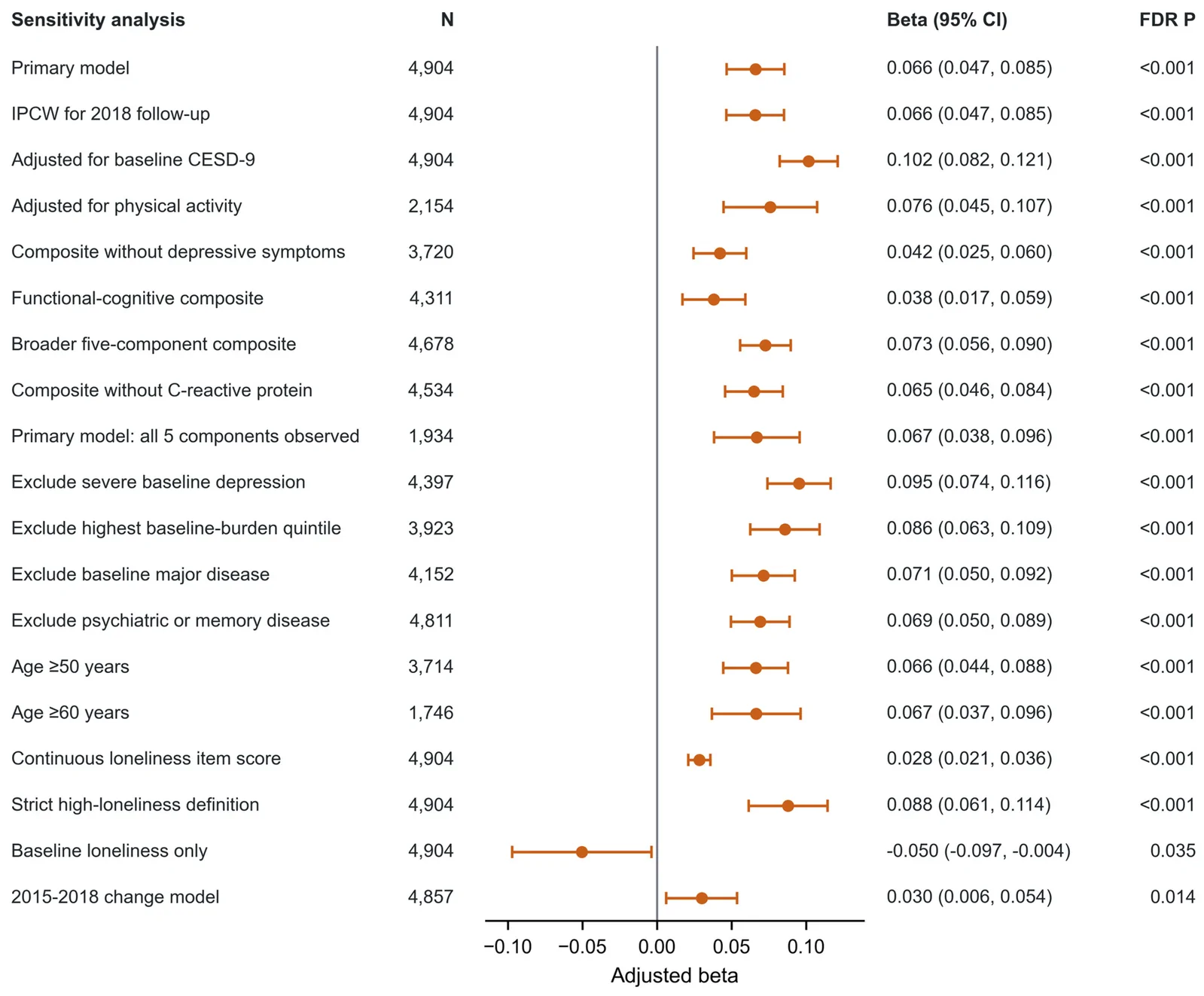
Methodological sensitivity analyses for assigned high-loneliness exposure and observation-interval burden. Points show adjusted beta coefficients, and bars show 95% confidence intervals. Most estimates are per two-year-scaled exposure unit; alternative exposure definitions retain their respective scales. The broader and depression/C-reactive protein (CRP)-exclusion composites contain no CKM stage contribution. The complete-component restriction fits the primary outcome among women with all five retained broader-composite components observed. The strict 2015–2018 model adjusts for CKM-free 2015 burden. Orange denotes FDR-adjusted *p* < 0.05 across all 19 specifications. Exact definitions are in Tables S6 and S28.

**Figure 6:**
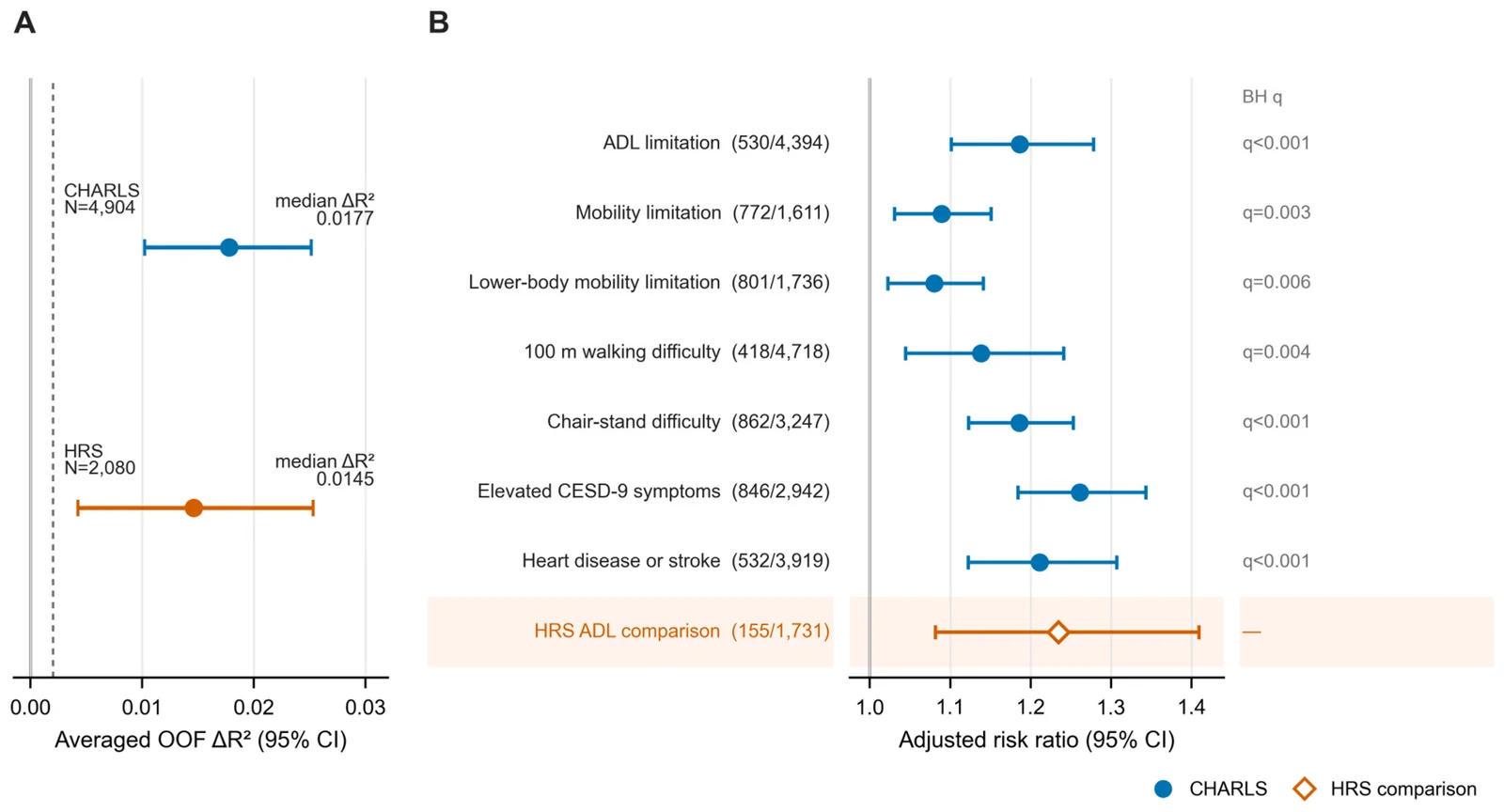
Incremental information and secondary incident risks. (**A**) Difference in averaged out-of-fold R-squared between repeated-measure and baseline-only models in women in CHARLS and HRS. Bars show paired participant-bootstrap 95% confidence intervals, and annotations show the median of ten paired per-seed differences. The dashed line marks the prespecified CHARLS ΔR^2^ criterion of 0.002. Because the outcome interval partly overlaps loneliness ascertainment, this panel quantifies descriptive incremental information rather than prospective clinical prediction. (**B**) Modified Poisson relative risks with 95% confidence intervals for all seven CHARLS 2015–2018 cumulative-exposure incident endpoints and the harmonized HRS incident-ADL comparison. Labels give events/participants. Benjamini–Hochberg q values apply only to the seven CHARLS endpoints; the dash indicates that HRS was outside this family. CHARLS relative risks are per two assigned high-loneliness years, whereas the HRS relative risk is per one additional high-loneliness assessment. HRS is a harmonized comparison, not independent validation, and neither panel estimates a causal effect. Blue denotes CHARLS and orange denotes the HRS comparison.

The adjusted continuous-exposure estimate remained positive when depressive symptoms were excluded (β = 0.042, 95% CI 0.025–0.060) and for the functional-cognitive composite (β = 0.038, 95% CI 0.017–0.059), as well as across several restrictions (Figure 5; Supplementary Tables S5, S6, S10 and S11; Supplementary Figure S1). Equal weighting of the three loneliness waves gave a similar standardized primary estimate (β = 0.057, 95% CI 0.038–0.076) to the duration-weighted score (β = 0.064, 95% CI 0.045–0.083), with concordant domain results (Supplementary Table S26). In women, associations were similar at ages 45–59 and 60–74; the ≥75 estimate was imprecise, and the age-group interaction was null (*p* = 0.607; Supplementary Table S25). In paired repeated five-fold cross-validation, adding later assessments produced statistically detectable but small gains in women in CHARLS (median ΔR^2^ = 0.0177; averaged ΔR^2^ = 0.0178, 95% CI 0.0103–0.0254) and men (median ΔR^2^ = 0.0146; averaged ΔR^2^ = 0.0148, 95% CI 0.0067–0.0228). These cross-validation comparisons are shown in Figure 6A and Supplementary Table S23. The cumulative-exposure estimates were nearly identical in women and men, and the CHARLS sex interaction was null (*p* = 0.597; Supplementary Tables S24 and S25).

In HRS, repeated measures produced a modest gain in women (median ΔR^2^ = 0.0145; averaged ΔR^2^ = 0.0146, 95% CI 0.0040–0.0250) and a smaller, imprecise gain in men (median ΔR^2^ = 0.0031; averaged ΔR^2^ = 0.0029, 95% CI −0.0072–0.0124). Cumulative-exposure associations were positive in both sexes, with no HRS sex interaction (*p* = 0.587; Supplementary Tables S24 and S25). The primary female comparisons are reported in Supplementary Tables S12 and S13. UK Biobank analyses were restricted to women and concerned baseline measurements only. The joint loneliness and infrequent-confiding indicator was associated with greater depressive symptoms, grip weakness, and a smaller inflammatory difference (Supplementary Table S14). This provides a cross-sectional comparison of related phenotypes, not confirmation of trajectories, incremental information, or prospective prediction.

The temporally separated analyses provided risk-scale estimates, but these were explicitly secondary and exploratory. Among 4394 women in CHARLS without ADL limitation in 2015 (530 incident cases), each two-year-scaled exposure unit was associated with an incident-ADL relative risk (RR) of 1.186 (95% CI 1.101 to 1.278). Covariate-standardized risk was 10.7% in the never-high group and 17.3% in the persistent group, a difference of 6.6 percentage points (95% CI 0.4 to 12.7). All seven cumulative-exposure endpoint estimates were positive in direction and had FDR-adjusted *p* < 0.05. HRS incident ADL gave RR = 1.235 (95% CI 1.082 to 1.409) per additional high-loneliness assessment. After the CKM-free reconstruction, the strict continuous 2015–2018 composite estimate adjusted for 2015 burden was small and positive (β = 0.030, 95% CI 0.006–0.054). Strict HRS continuous-change findings remained weak. Incremental incident-ADL prediction was negligible: the change in area under the receiver operating characteristic curve (AUC) was 0.0024 in CHARLS and 0.0007 in HRS, with both intervals crossing zero. These secondary exploratory incident results improve temporal separation for selected functional outcomes but do not provide confirmatory evidence for a generalized multisystem aging interpretation (Figure 6B; Supplementary Table S16). Detailed disease-specific, alternative-exposure, strict-change, and unadjusted specifications are reported in Supplementary Tables S15 and S17–S22.

## 4. Discussion

Repeated loneliness assessments provided information beyond baseline loneliness and health, but the gains were modest. In CHARLS, this additional information was detectable in both women and men; HRS showed a modest gain in women and an imprecise estimate in men. In the primary analysis in women, associations were concentrated in depressive symptoms and everyday function, with little evidence of a generalized multisystem pattern. This extends prior CHARLS trajectory work [23] and baseline disability analyses [24] by using fixed held-out comparisons. Related studies connect changing social disconnection with cognition [5] and cumulative loneliness with mortality and memory [1,42]. The comparison with baseline helps distinguish the presence of trajectory associations from the amount of information gained by measuring loneliness again.

The domain-specific findings clarify what contributed to the composite association. Loneliness has been associated with physical frailty [43], and reciprocal longitudinal associations between loneliness and frailty have been reported [44]. Prospective evidence also links chronic loneliness phenotypes to incident functional impairment [22] and persistent loneliness among women to dementia risk [45]. In CHARLS, depressive symptoms, ADL, mobility, grip, and the physical-function composite showed more consistent associations than metabolic or inflammatory markers. Proposed neurobiological and behavioral pathways [46,47] provide hypotheses, but the present data do not establish a sequence in which affective change causes functional loss and then biological dysregulation. These findings point to psychological and functional vulnerability rather than a common change across all measured systems.

The original focus on women reflected their psychological-health burden and social circumstances rather than an assumption that loneliness is uniquely harmful to women. Older women in CHARLS have been reported to have a higher burden of depressive symptoms [30], and studies of family relationships and coping resources indicate that psychosocial stress may be experienced in different social circumstances by women and men [29,31]. Evidence of greater mental-health deterioration among women during the pandemic is specific to that social disruption [32]; it does not establish a universal sex difference in responses to loneliness. We did not measure sex-specific stress mechanisms or hormonal pathways. The added analyses in men therefore address an important limit of the original sample: cumulative-exposure coefficients were nearly identical in women and men in CHARLS, and neither CHARLS nor HRS showed a clear sex interaction. Prior HRS work has reported sex differences for memory outcomes [42], but such findings cannot be generalized to every domain.

The broad age range addresses the information gained from repeated assessment across midlife and older age rather than a shared cause of loneliness. Loneliness varies nonlinearly across adulthood [48]. Work and caregiving demands may shape loneliness differently from retirement, bereavement or dependence on assistance. In particular, expected help or financial transfers from children may have a different meaning for an employed person in midlife than for someone needing support in later life. These are possible contexts, not mechanisms tested by our models. The existing estimates were similar at ages 45–59 and 60–74, but the ≥75-year estimate was imprecise. The null age-group interaction does not establish uniform associations or social mechanisms across ages. Similarly, the sex-interaction results do not establish equivalence between women and men. The pooled findings should therefore be read alongside the subgroup estimates, with caution for the oldest participants.

The cross-cohort comparisons place this domain-specific pattern in context. Psychological and functional associations were the clearest common findings, and both longitudinal cohorts showed small gains from repeated assessment in women. The estimate in men in HRS was less precise. CHARLS included physical changes ending in either 2015 or 2018, whereas the final HRS follow-up composite contained depressive symptoms, ADL limitations, and cognition. UK Biobank used a joint loneliness and infrequent-confiding indicator and baseline outcomes only. Differences in age, selection, measurement, and follow-up prevent direct comparison of coefficient magnitudes or attribution of differences to nationality. Cross-national trajectory research identifies both country variation and recurring correlates such as living alone and widowhood [49]. A 24-country study including CHARLS and HRS also associated the joint pattern of socioeconomic disadvantage, social inactivity, and loneliness with subsequent depressive symptoms [50]. These findings support examining loneliness in its social context while leaving the contribution of culture unresolved. Expectations about support from children, family relationships, and social participation may therefore be relevant to interpretation, but these cultural mechanisms were not tested here.

Women whose loneliness remitted had a lower-burden pattern, but they also entered follow-up with a more favorable baseline profile. Longitudinal evidence supports reciprocal temporal relations between loneliness and frailty or social isolation [44,51]. The adjusted remission contrast describes this observational pattern and does not estimate the effect of reducing loneliness.

The clinical implications require a distinction between added statistical information and improved health outcomes. Some psychosocial and behavioral trials have modified loneliness, although effect sizes and study quality vary [52,53,54]. Those trials do not establish that changing loneliness prevents later functional or cognitive outcomes. Prediction performance, calibration and decision utility address different questions [38]. Although the fixed incremental-information criterion was met, clinical use would require independent evaluation of transportability, absolute-risk calibration, decision-curve benefit, and prospective implementation.

Temporal overlap is particularly important for interpretation. Changes in health during 2011–2015 may influence later loneliness responses as well as contribute to the primary outcome. Removing the loneliness item from CES-D reduces direct item duplication but not common reporting influences or shared affective content. Baseline adjustment cannot remove time-varying confounding or reverse causation, and cross-validation cannot establish that exposure precedes outcome. The secondary incident analyses restricted participants to those free of each endpoint in 2015 and assessed onset by 2018. These improve temporal separation from the measured loneliness history, although the duration-weighted score still assigns the 2015 status to the following interval. The temporally separated analyses also showed positive associations with incident ADL limitation in CHARLS and HRS. The revised strict 2015–2018 continuous-change estimate was small and positive, whereas incident-ADL discrimination gains were negligible. The strict-change result was recalculated after the post hoc removal of CKM-dependent scoring and should be interpreted as a sensitivity analysis, not independent confirmation. These analyses extend the descriptive findings to selected subsequent functional outcomes but provide limited evidence of useful prospective prediction.

Several further limitations constrain inference. CHARLS used one direct CES-D loneliness item, whereas HRS used an indirect three-item scale. A single item cannot provide an internal-consistency estimate and may be more vulnerable to random misclassification; population evidence shows incomplete convergence with longer measures [55]. The 2:2:3 score assigns observed status to unequal intervals. Equal weighting gave similar estimates but cannot verify within-interval stability.

Population health also changed during the broader period, with increases in both life expectancy and health-adjusted life expectancy in China [56]. These national trends provide context rather than a measured exposure in this study. Changes in health and survival, and in the circumstances of family support, could influence who remained under observation and how loneliness related to health burden. The CHARLS analysis covered seven years, not a fifteen-year follow-up. Because participants were observed over the same survey waves, our current models could not disentangle aging, birth-cohort and calendar-period influences. Baseline age and health adjustment did not estimate the effect of changing healthy life expectancy. We therefore cannot quantify how much secular change contributed to the associations or assume that the same estimates apply to later periods.

The adjusted primary association was absent in unadjusted models, making baseline adjustment and residual imbalance central to interpretation. Complete observation selected 4904 women from 7980 baseline-eligible women. The male and interaction analyses were post hoc; the oldest stratum and HRS male estimates were imprecise. The composite was internally constructed, covariate fills and available-component scoring may affect estimates, and biomarker samples were smaller. Neither comparison cohort provided independent validation of the CHARLS model. During revision, we excluded CKM-derived secondary measures whose documentation did not provide the full component rules needed for transparent reporting. The affected analyses were repeated, but the limitations of the remaining observational measures persist.

## 5. Conclusions

Repeated loneliness histories provided statistically detectable but small incremental information beyond baseline status in women and men in CHARLS, with more variable HRS gains. The primary analysis in women identified vulnerability concentrated in depressive symptoms and everyday function, a smaller cognitive association, and little evidence of associations with biomarkers. Sex and age interactions were not supported, and equal weighting did not materially change the findings. The findings therefore support repeated assessment as a way to characterize psychological and functional burden more fully. Overlapping measurement windows and dependence on baseline adjustment limit causal interpretation, while the post hoc sex analyses and limited time-separated evidence leave generalized-aging and clinical-prediction interpretations unestablished.

## Data Availability

The data presented in this study are available on request from the corresponding author. The data are not publicly available due to restrictions associated with the UK Biobank, CHARLS, and HRS data-use agreements governing access to these cohorts.

## Supplementary Materials

The following supporting information can be downloaded at: https://www.mdpi.com/article/10.3390/healthcare14183058/s1, Figure S1, Data-driven continuous loneliness-trajectory sensitivity analysis; Tables S1–S30, study flow, descriptive characteristics, primary models, sensitivity analyses, cross-cohort comparisons, incident-outcome analyses, attrition analyses, paired cross-validation performance, sex and age comparisons, equal-weight sensitivity analyses, and cohort-specific sample and measurement definitions. The cohort-profile and measurement sources cited in Tables S27 and S29 are included in the main reference list [28,33,34,41,57,58].

## Abbreviations

ADL, activities of daily living; AUC, area under the receiver operating characteristic curve; CES-D, Center for Epidemiologic Studies Depression Scale; CHARLS, China Health and Retirement Longitudinal Study; FDR, false discovery rate; HRS, Health and Retirement Study; IADL, instrumental activities of daily living; IPCW, inverse-probability-of-censoring weighting; MAE, mean absolute error; RMSE, root mean squared error.

## References

[1] Yu X., Cho T.-C., Westrick A.C., Chen C., Langa K.M., Kobayashi L.C. (2023). Association of cumulative loneliness with all-cause mortality among middle-aged and older adults in the United States, 1996 to 2019. Proc. Natl. Acad. Sci. USA.

[2] Meng L., Xu R., Li J., Hu J., Xu H., Wu D., Hu X., Zeng X., Zhang Q., Li J. (2024). The silent epidemic: Exploring the link between loneliness and chronic diseases in China’s elderly. BMC Geriatr..

[3] Cacioppo J.T., Hughes M.E., Waite L.J., Hawkley L.C., Thisted R.A. (2006). Loneliness as a specific risk factor for depressive symptoms: Cross-sectional and longitudinal analyses. Psychol. Aging.

[4] Perissinotto C.M., Stijacic Cenzer I., Covinsky K.E. (2012). Loneliness in older persons: A predictor of functional decline and death. Arch. Intern. Med..

[5] Huang Q.-M., Zhang P.-D., Shen D., Gao J., Li Z.-H., Lv Y.-B., Shi X.-M., Mao C. (2024). Analysis of changes in social isolation, loneliness, or both, and subsequent cognitive function among older adults: Findings from a nationwide cohort study. Alzheimers Dement..

[6] The Lancet (2023). Loneliness as a health issue. Lancet.

[7] Miao M.-Y., Fang F., Lyu J.-Q., Liu Z.-Y., Wan Z.-X., Qin L.-Q., Chen G.-C., Wang H.-P. (2025). Relationships of social isolation and loneliness with healthy aging among older adults. BMC Geriatr..

[8] Barton S., Zovko A., Müller C., Krabichler Q., Schulze J., Wagner S., Grinevich V., Shamay-Tsoory S., Hurlemann R. (2024). A translational neuroscience perspective on loneliness: Narrative review focusing on social interaction, illness and oxytocin. Neurosci. Biobehav. Rev..

[9] Bowirrat A., Elman I., Dennen C.A., Gondré-Lewis M.C., Cadet J.L., Khalsa J., Baron D., Soni D., Gold M.S., McLaughlin T.J. (2023). Neurogenetics and epigenetics of loneliness. Psychol. Res. Behav. Manag..

[10] Blazer D. (2025). Loneliness and solitude: The yin and yang of social disconnection. Am. J. Geriatr. Psychiatry.

[11] Taylor H.O., Cudjoe T.K.M., Bu F., Lim M.H. (2023). The state of loneliness and social isolation research: Current knowledge and future directions. BMC Public Health.

[12] Aartsen M., Vangen H., Pavlidis G., Hansen T., Precupetu I. (2024). The unique and synergistic effects of social isolation and loneliness on 20-years mortality risks in older men and women. Front. Public Health.

[13] Kuiper J.S., Zuidersma M., Oude Voshaar R.C., Zuidema S.U., van den Heuvel E.R., Stolk R.P., Smidt N. (2015). Social relationships and risk of dementia: A systematic review and meta-analysis of longitudinal cohort studies. Ageing Res. Rev..

[14] Holt-Lunstad J., Steptoe A. (2022). Social isolation: An underappreciated determinant of physical health. Curr. Opin. Psychol..

[15] Hong J.H., Nakamura J.S., Berkman L.F., Chen F.S., Shiba K., Chen Y., Kim E.S., VanderWeele T.J. (2023). Are loneliness and social isolation equal threats to health and well-being? An outcome-wide longitudinal approach. SSM Popul. Health.

[16] Nakagomi A., Tsuji T., Saito M., Ide K., Kondo K., Shiba K. (2023). Social isolation and subsequent health and well-being in older adults: A longitudinal outcome-wide analysis. Soc. Sci. Med..

[17] Mund M., Freuding M.M., Möbius K., Horn N., Neyer F.J. (2020). The stability and change of loneliness across the life span: A meta-analysis of longitudinal studies. Pers. Soc. Psychol. Rev..

[18] Di Gessa G., Bordone V., Arpino B. (2025). Trajectories of loneliness in later life—Evidence from a 10-year English panel study. Soc. Sci. Med..

[19] Li X., Wu C., Feng T., Chen X., Chen H., Lu X., Gao J., Hou C. (2025). Loneliness trajectories in older adults: A systematic review. Arch. Gerontol. Geriatr..

[20] Domènech-Abella J., Gabarrell-Pascuet A., Mundó J., Haro J.M., Varga T.V. (2024). Chronic and Transient Loneliness in Western Countries: Risk Factors and Association With Depression. A 2-Year Follow-Up Study. Am. J. Geriatr. Psychiatry.

[21] HaGani N., Owen K., Clare P.J., Merom D., Smith B.J., Ding D. (2025). Long-term elevated levels of loneliness are linked to lower health-related quality of life in middle-aged Australian women. Commun. Psychol..

[22] Gao Q., Steptoe A., Fancourt D. (2025). Chronic loneliness and isolation phenotypes, incident functional impairment and mortality in England between 2004 and 2018. Nat. Ment. Health.

[23] Huang Y., Zhu X., Liu X., Li J. (2024). Loneliness trajectories predict risks of cardiovascular diseases in Chinese middle-aged and older adults. J. Gerontol. B Psychol. Sci. Soc. Sci..

[24] Guo L., An L., Luo F., Yu B. (2021). Social isolation, loneliness and functional disability in Chinese older women and men: A longitudinal study. Age Ageing.

[25] Lee S.L., Pearce E., Ajnakina O., Johnson S., Lewis G., Mann F., Pitman A., Solmi F., Sommerlad A., Steptoe A. (2021). The association between loneliness and depressive symptoms among adults aged 50 years and older: A 12-year population-based cohort study. Lancet Psychiatry.

[26] Bull L.M., Lunt M., Martin G.P., Hyrich K., Sergeant J.C. (2020). Harnessing repeated measurements of predictor variables for clinical risk prediction: A review of existing methods. Diagn. Progn. Res..

[27] Moons K.G.M., Kengne A.P., Woodward M., Royston P., Vergouwe Y., Altman D.G., Grobbee D.E. (2012). Risk prediction models: I. Development, internal validation, and assessing the incremental value of a new (bio)marker. Heart.

[28] Zhao Y., Hu Y., Smith J.P., Strauss J., Yang G. (2014). Cohort profile: The China Health and Retirement Longitudinal Study (CHARLS). Int. J. Epidemiol..

[29] Chauhan S., Carr D.C., Taylor M. (2025). Associations between pre-widowhood psychological resilience and subsequent depressive symptom recovery following spousal loss among men and women. Gerontologist.

[30] Li H., Liu X., Zheng Q., Zeng S., Luo X. (2022). Gender differences and determinants of late-life depression in China: A cross-sectional study based on CHARLS. J. Affect. Disord..

[31] Cheung E.S.L., Mui A.C. (2023). Gender variation and late-life depression: Findings from a national survey in the USA. Ageing Int..

[32] Zaninotto P., Iob E., Demakakos P., Steptoe A. (2022). Immediate and Longer-Term Changes in the Mental Health and Well-being of Older Adults in England During the COVID-19 Pandemic. JAMA Psychiatry.

[33] Sonnega A., Faul J.D., Ofstedal M.B., Langa K.M., Phillips J.W., Weir D.R. (2014). Cohort profile: The Health and Retirement Study (HRS). Int. J. Epidemiol..

[34] Sudlow C., Gallacher J., Allen N., Beral V., Burton P., Danesh J., Downey P., Elliott P., Green J., Landray M. (2015). UK Biobank: An open access resource for identifying the causes of a wide range of complex diseases of middle and old age. PLoS Med..

[35] Yu X., Jones R.N., Kobayashi L.C., Gross A.L. (2025). Cross-national statistical harmonization of the Center for Epidemiologic Studies Depression (CES-D) scale among older adults in China, England, India, Mexico, South Africa, and the United States. J. Clin. Epidemiol..

[36] MacKinnon J.G., White H. (1985). Some heteroskedasticity-consistent covariance matrix estimators with improved finite sample properties. J. Econom..

[37] Benjamini Y., Hochberg Y. (1995). Controlling the false discovery rate: A practical and powerful approach to multiple testing. J. R. Stat. Soc. B.

[38] Steyerberg E.W., Vickers A.J., Cook N.R., Gerds T., Gonen M., Obuchowski N., Pencina M.J., Kattan M.W. (2010). Assessing the performance of prediction models: A framework for traditional and novel measures. Epidemiology.

[39] Zou G. (2004). A modified Poisson regression approach to prospective studies with binary data. Am. J. Epidemiol..

[40] Lee J., Phillips D., Wilkens J., Gateway to Global Aging Data Team (2021). Gateway to Global Aging Data: Resources for cross-national comparisons of family, social environment, and healthy aging. J. Gerontol. B Psychol. Sci. Soc. Sci..

[41] Hughes M.E., Waite L.J., Hawkley L.C., Cacioppo J.T. (2004). A short scale for measuring loneliness in large surveys: Results from two population-based studies. Res. Aging.

[42] Yu X., Westrick A.C., Kobayashi L.C. (2023). Cumulative loneliness and subsequent memory function and rate of decline among adults aged 50 years or older in the United States, 1996 to 2016. Alzheimers Dement..

[43] Kojima G., Taniguchi Y., Aoyama R., Tanabe M. (2022). Associations between loneliness and physical frailty in community-dwelling older adults: A systematic review and meta-analysis. Ageing Res. Rev..

[44] Ye L., Bally E., Korenhof S.A., Fierloos I., Alhambra Borrás T., Clough G., Raat H., van Grieken A. (2024). The association between loneliness and frailty among community-dwelling older adults in five European countries: A longitudinal study. Age Ageing.

[45] Htun H.L., Teshale A.B., Sun H., Ryan J., Owen A.J., Woods R.L., Shah R.C., Chong T.T.-J., Freak-Poli R. (2025). Changes in loneliness, social isolation, and social support: A gender-disaggregated analysis of their associations with dementia and cognitive decline in older adults. Int. J. Geriatr. Psychiatry.

[46] Dabiri S., Mwendwa D.T., Campbell A. (2024). Psychological and neurobiological mechanisms involved in the relationship between loneliness and cognitive decline in older adults. Brain Behav. Immun..

[47] Du C., Li X., Li J., Wang W., Dang M., Cheng J., Xu K., Wang J., Chen C., Chen Y. (2024). Leisure activities as reserve mediators of the relationship between loneliness and cognition in aging. Transl. Psychiatry.

[48] Graham E.K., Beck E.D., Jackson K., Yoneda T., McGhee C., Pieramici L., Atherton O.E., Luo J., Willroth E.C., Steptoe A. (2024). Do we become more lonely with age? A coordinated data analysis of nine longitudinal studies. Psychol. Sci..

[49] Sheftel M.G., Margolis R., Verdery A.M. (2024). Loneliness trajectories and chronic loneliness around the world. J. Gerontol. B Psychol. Sci. Soc. Sci..

[50] Wang Y., Liu M., Yang F., Chen H., Wang Y., Liu J. (2024). The associations of socioeconomic status, social activities, and loneliness with depressive symptoms in adults aged 50 years and older across 24 countries: Findings from five prospective cohort studies. Lancet Healthy Longev..

[51] Mehrabi F., Pomeroy M.L., Cudjoe T.K.M., Jenkins E., Dent E., Hoogendijk E.O. (2024). The temporal sequence and reciprocal relationships of frailty, social isolation and loneliness in older adults across 21 years. Age Ageing.

[52] Hoang P., King J.A., Moore S., Moore K., Reich K., Sidhu H., Tan C.V., Whaley C., McMillan J. (2022). Interventions associated with reduced loneliness and social isolation in older adults: A systematic review and meta-analysis. JAMA Netw. Open.

[53] Gilbody S., Littlewood E., McMillan D., Atha L., Bailey D., Baird K., Brady S., Burke L., Chew-Graham C.A., Coventry P. (2024). Behavioural activation to mitigate the psychological impacts of COVID-19 restrictions on older people in England and Wales (BASIL+): A pragmatic randomised controlled trial. Lancet Healthy Longev..

[54] Kwok J.Y.Y., Jiang D., Yeung D.Y.-L., Choi N.G., Ho R.T.H., Warner L.M., Chou K.-L. (2024). Layperson-delivered telephone-based behavioral activation among low-income older adults during the COVID-19 pandemic: The HEAL-HOA randomized clinical trial. JAMA Netw. Open.

[55] Reinwarth A.C., Ernst M., Krakau L., Brähler E., Beutel M.E. (2023). Screening for loneliness in representative population samples: Validation of a single-item measure. PLoS ONE.

[56] Chen L., Wang L., Qian Y., Chen H. (2022). Changes and trend disparities in life expectancy and health-adjusted life expectancy attributed to disability and mortality from 1990 to 2019 in China. Front. Public Health.

[57] Smith J., Ryan L., Larkina M., Sonnega A., Weir D. (2023). Psychosocial and Lifestyle Questionnaire 2006–2022: User Guide.

[58] RAND Corporation RAND HRS Data Products. https://www.rand.org/well-being/social-and-behavioral-policy/centers/aging/dataprod/hrs-data.html.

