# Supplementary material for: Incremental Information from Repeated Loneliness Assessments in Middle-Aged and Older Adults
Source: Healthcare (Basel). 2026 Sep 17;14(18):3058. doi: 10.3390/healthcare14183058 (PMC13606363; doi:10.3390/healthcare14183058)
Supplement: Supplementary file 1 [file healthcare-14-03058-s001.zip › healthcare-4522400-supplementary.pdf]

Supplementary Materials

Incremental Information from Repeated Loneliness Assessments in Middle-Aged and Older Adults

Zhengqi Qiu, Yunzhi Emma Huang, Juntao Kan, Lin Xu

Contents

Supplementary Table S1. Study population flow
Supplementary Table S2. Baseline characteristics by loneliness-trajectory group
Supplementary Table S3. Loneliness trajectories from 2011 to 2015 and 2011–2018 observation-interval burden
Supplementary Table S4. Two-year-scaled assigned high-loneliness exposure and domain-specific outcomes. A one-unit increase approximates two assigned high-loneliness years.
Supplementary Table S5. Inverse-probability-of-censoring weighted trajectory model
Supplementary Table S6. Full sensitivity analyses
Supplementary Table S7. Correlates of loneliness remission among women lonely at baseline
Supplementary Table S8. Baseline profile of remitted and non-remitted women with high loneliness at baseline
Supplementary Table S9. Observation-interval burden contrast among women with high loneliness at baseline
Supplementary Table S10. Subgroup analyses for two-year-scaled high-loneliness exposure
Supplementary Table S11. Data-driven continuous loneliness-trajectory sensitivity analysis
Supplementary Table S12. HRS harmonized longitudinal trajectory comparison
Supplementary Table S13. HRS domain-specific outcome comparison
Supplementary Table S14. UK Biobank baseline phenotype convergence

Supplementary Table S15. CHARLS disease-specific confounding adjustment.
Supplementary Table S16. Secondary endpoint-eligible CHARLS 2015–2018 incident-outcome models.
Supplementary Table S17. CHARLS 2015–2018 continuous change-outcome models.
Supplementary Table S18. HRS wave 12–13 post-exposure change models.
Supplementary Table S19. HRS disease-specific confounding adjustment.
Supplementary Table S20. Unadjusted CHARLS trajectory and domain models.
Supplementary Table S21. Unadjusted HRS comparison models.
Supplementary Table S22. Early attrition and trajectory-completion analyses.
Supplementary Table S23. Fixed paired cross-validation performance for single-wave and repeated-loneliness models
Supplementary Table S24. Sex-stratified incremental-information sensitivity analyses.
Supplementary Table S25. Sex- and age-stratified association sensitivity analyses.
Supplementary Table S26. Equal-weight sensitivity analyses across outcome domains.
Supplementary Table S27. Cohort roles and analytic sample characteristics

Supplementary Table S28. CHARLS exposure outcome and covariate definitions

Supplementary Table S29. HRS exposure outcome and covariate definitions

Supplementary Table S30. UK Biobank baseline exposure outcome and covariate definitions

Supplementary Figure S1. Data-driven continuous loneliness-trajectory sensitivity analysis

Supplementary Table S1. Study population flow.

Flow step	Included participants, n	Excluded since previous step, n
Enhanced CHARLS analytic source dataset	25,586	
Female participants	13,244	12,342
Female participants aged 45 years or older	8,663	4,581
Complete loneliness trajectory in 2011, 2013, and 2015	5,677	2,986
Nonmissing aligned baseline burden score	5,672	5
Final primary sample with 2011–2018 observation-interval burden composite	4,904	768

Note. Counts are sequential. Each exclusion is calculated from the immediately preceding step.

Supplementary Table S2. Baseline characteristics by loneliness-trajectory group.

Baseline characteristic	Available N	Overall	Never-high loneliness	Remitted loneliness	Incident loneliness	Intermittent loneliness	Persistent loneliness	P value across trajectories
Participants, n	4904	4,904	3,108	421	879	321	175	
Age, years, mean (SD)	4904	56.9 (8.2)	56.3 (7.9)	57.7 (8.8)	57.8 (8.3)	58.8 (8.8)	58.8 (7.6)	<0.001
Rural residence, n (%)	4904	3,126 (63.7)	1,850 (59.5)	302 (71.7)	606 (68.9)	230 (71.7)	138 (78.9)	<0.001
Low education	4904	2,818 (57.5)	1,614 (51.9)	277 (65.8)	596 (67.8)	209 (65.1)	122 (69.7)	<0.001
Middle education	4904	1,717 (35.0)	1,218 (39.2)	119 (28.3)	241 (27.4)	91 (28.3)	48 (27.4)	
High education	4904	369 (7.5)	276 (8.9)	25 (5.9)	42 (4.8)	21 (6.5)	5 (2.9)	
Body mass index, kg/m2, mean (SD)	4904	24.0 (3.8)	24.3 (3.8)	23.7 (3.6)	23.6 (3.8)	23.5 (3.9)	23.2 (3.2)	<0.001
Ever smoked, n (%)	4904	359 (7.3)	174 (5.6)	37 (8.8)	94 (10.7)	31 (9.7)	23 (13.1)	<0.001
Alcohol drinking in last year, n (%)	4904	599 (12.2)	349 (11.2)	68 (16.2)	99 (11.3)	55 (17.1)	28 (16.0)	<0.001
Not partnered, n (%)	4904	595 (12.1)	234 (7.5)	88 (20.9)	127 (14.4)	79 (24.6)	67 (38.3)	<0.001
No social activity, n (%)	4903	2,594 (52.9)	1,574 (50.6)	239 (56.9)	497 (56.5)	180 (56.1)	104 (59.4)	0.001
Social isolation index, mean (SD)	4904	0.7 (0.6)	0.6 (0.6)	0.8 (0.6)	0.7 (0.6)	0.8 (0.6)	1.0 (0.7)	<0.001

Baseline characteristic	Available N	Overall	Never-high loneliness	Remitted loneliness	Incident loneliness	Intermittent loneliness	Persistent loneliness	P value across trajectories
Number of chronic conditions, mean (SD)	4904	1.1 (1.1)	1.0 (1.1)	1.2 (1.2)	1.2 (1.1)	1.4 (1.3)	1.6 (1.2)	<0.001
Baseline diabetes, n (%)	4858	300 (6.2)	188 (6.1)	23 (5.5)	62 (7.2)	22 (6.9)	5 (2.9)	0.249
Baseline heart disease, stroke, or cancer, n (%)	4904	752 (15.3)	401 (12.9)	77 (18.3)	163 (18.5)	73 (22.7)	38 (21.7)	<0.001
CES-D-9 score excluding loneliness item, mean (SD)	4904	8.7 (6.0)	6.8 (5.0)	12.8 (6.0)	9.6 (5.6)	14.6 (5.9)	16.8 (5.8)	<0.001
Global cognitive score, mean (SD)	4303	13.9 (5.0)	14.7 (5.0)	12.6 (4.7)	12.9 (4.8)	12.3 (4.9)	11.9 (4.6)	<0.001
Grip strength, kg, mean (SD)	4242	26.8 (7.0)	27.4 (6.9)	25.8 (7.2)	25.9 (7.1)	25.5 (7.2)	24.8 (8.2)	<0.001
ADL limitations, mean (SD)	4856	0.2 (0.6)	0.1 (0.5)	0.2 (0.7)	0.2 (0.6)	0.4 (0.9)	0.5 (1.0)	<0.001
Aligned baseline burden score, mean (SD)	4904	0.2 (0.6)	0.0 (0.5)	0.5 (0.6)	0.3 (0.5)	0.7 (0.7)	0.8 (0.7)	<0.001

Supplementary Table S3. Loneliness trajectories from 2011 to 2015 and 2011–2018 observation-interval burden.

Loneliness trajectory (2011–2015)	Operational definition	Participants, n (%)	Adjusted beta (95% CI)	P value	FDR-adjusted P value
Never high loneliness	Low in 2011, 2013, and 2015	3,108 (63.4)	Reference		
Remitted loneliness	High in 2011; low in 2013 and 2015	421 (8.6)	-0.083 (-0.144, -0.021)	0.009	0.012
Incident loneliness	Low in 2011; high in 2013 and/or 2015	879 (17.9)	0.191 (0.146, 0.236)	<0.001	<0.001
Intermittent loneliness	High in 2011 and one later wave	321 (6.5)	0.043 (-0.031, 0.118)	0.255	0.255
Persistent loneliness	High in 2011, 2013, and 2015	175 (3.6)	0.178 (0.079, 0.276)	<0.001	<0.001

Supplementary Table S4. Two-year-scaled assigned high-loneliness exposure and domain-specific outcomes. A one-unit increase approximates two assigned high-loneliness years.

Outcome domain	Outcome	Effect scale	Participants, n	Events, n	Estimate (95% CI)	P value	FDR-adjusted P value
Overall	Primary psychological-functional-cognitive observation-interval burden	Adjusted beta	4904	-	0.066 (0.047, 0.085)	<0.001	<0.001
Psychological	Depressive symptoms worsening (CES-D-9)	Adjusted beta	4851	-	0.201 (0.171, 0.232)	<0.001	<0.001
Physical function	ADL limitations worsening	Adjusted beta	4855	-	0.084 (0.053, 0.115)	<0.001	<0.001
Physical function	Physical performance composite worsening	Adjusted beta	4860	-	0.055 (0.034, 0.077)	<0.001	<0.001
Physical function	Mobility limitations worsening	Adjusted beta	4860	-	0.058 (0.023, 0.094)	0.001	0.002
Physical function	Grip strength decline	Adjusted beta	3684	-	0.047 (0.021, 0.073)	<0.001	<0.001
Cognitive	Global cognitive score decline	Adjusted beta	3701	-	0.047 (0.015, 0.079)	0.004	0.006
Physical function	Walking-test time increase	Adjusted beta	1374	-	0.015 (-0.033, 0.064)	0.532	0.650
Physical function	Chair-stand slowing	Adjusted beta	3652	-	0.000 (-0.037, 0.038)	0.988	0.988
Metabolic biomarkers	Metabolic biomarker deterioration	Adjusted beta	3026	-	-0.004 (-0.021, 0.013)	0.619	0.681
Inflammation	C-reactive protein increase	Adjusted beta	3026	-	0.014 (-0.015, 0.044)	0.345	0.474

Note. Cardiometabolic-stage-derived outcomes were excluded. Physical-function, mobility and metabolic models use baseline burden from cognition, grip, ADL, CES-D-9 and log-CRP, requiring at least three components. The primary model retains its aligned baseline score. FDR correction covers the 11 displayed outcomes.

Supplementary Table S5. Inverse-probability-of-censoring weighted trajectory model.

**Loneliness trajectory (2011–2015)**	**Adjusted beta (95% CI)**	**P value**
Never high loneliness	Reference	
Remitted loneliness	-0.077 (-0.141, -0.014)	0.016
Incident loneliness	0.191 (0.146, 0.236)	<0.001
Intermittent loneliness	0.042 (-0.033, 0.117)	0.269
Persistent loneliness	0.176 (0.076, 0.275)	<0.001

Note. IPCW indicates inverse-probability-of-censoring weighting for observation at the 2018 outcome wave.

Supplementary Table S6. Full sensitivity analyses.

Analysis	Participants, n	Exposure or model change	Adjusted beta (95% CI)	P value	FDR-adjusted P value
Primary model	4,904	Main two-year-scaled high-loneliness exposure based on repeated 2011, 2013, and 2015 assessments, with 2015 status carried forward to 2018; primary outcome excludes 2011–2015 biomarker changes.	0.066 (0.047, 0.085)	<0.001	<0.001
Inverse-probability weighting for 2018 follow-up	4,904	Inverse-probability-of-censoring weighted model.	0.066 (0.047, 0.085)	<0.001	<0.001
Additional adjustment for baseline CES-D-9	4,904	CES-D-9 excludes the loneliness item.	0.102 (0.082, 0.121)	<0.001	<0.001
Additional adjustment for physical activity	2,154	Behavioral confounding sensitivity.	0.076 (0.045, 0.107)	<0.001	<0.001
Composite score excluding depressive symptoms	3,720	Mean standardized cognition, grip, ADL and log-CRP changes; at least three of four required. Adjusted for the corresponding non-depressive baseline burden.	0.042 (0.025, 0.060)	<0.001	<0.001
Functional-cognitive composite only	4,311	Mean standardized cognition, grip and ADL changes; at least two of three required. Adjusted for non-depressive baseline burden, which also includes log-CRP.	0.038 (0.017, 0.059)	<0.001	<0.001
Broader five-component composite	4,678	Mean standardized cognition, grip, ADL, CES-D-9 and log-CRP changes; at least three of five required. Adjusted for corresponding baseline burden. Biomarker changes overlap the exposure window.	0.073 (0.056, 0.090)	<0.001	<0.001
Composite without C-reactive protein	4,534	Mean standardized cognition, grip, ADL and CES-D-9 changes; at least three of four required. Adjusted for five-component baseline burden, including log-CRP.	0.065 (0.046, 0.084)	<0.001	<0.001
Primary model with all five change components observed	1,934	Primary outcome and aligned baseline adjustment, restricted to observed changes in all five components: cognition, grip, ADL, CES-D-9 and log-CRP.	0.067 (0.038, 0.096)	<0.001	<0.001
Excluding severe baseline depressive symptoms	4,397	Reduces reverse causation by pre-existing depressive burden.	0.095 (0.074, 0.116)	<0.001	<0.001
Excluding highest baseline burden quintile	3,923	Reduces reverse causation by pre-existing burden.	0.086 (0.063, 0.109)	<0.001	<0.001
Excluding baseline heart disease, stroke, or cancer	4,152	Reduces confounding by major baseline disease.	0.071 (0.050, 0.092)	<0.001	<0.001
Excluding baseline psychiatric or memory disease	4,811	Reduces reverse causation by diagnosed mental or memory conditions.	0.069 (0.050, 0.089)	<0.001	<0.001
Restricting analysis to age 50 years or older	3,714	Tests whether inference depends on the CHARLS age-45 sampling threshold.	0.066 (0.044, 0.088)	<0.001	<0.001
Restricting analysis to age 60 years or older	1,746	Tests robustness in conventionally older women.	0.067 (0.037, 0.096)	<0.001	<0.001
Continuous loneliness item score	4,904	Uses continuous loneliness item scores rather than dichotomized high loneliness.	0.028 (0.021, 0.036)	<0.001	<0.001
Strict high-loneliness definition	4,904	Defines high loneliness as the highest item category only.	0.088 (0.061, 0.114)	<0.001	<0.001
Baseline loneliness only	4,904	Single baseline measurement rather than dynamic exposure.	-0.050 (-0.097, -0.004)	0.035	0.035
2015–2018 change adjusted for 2015 burden	4,857	2015–2018 cognition, ADL and CES-D-9 change composite, adjusted for 2015 cognition, grip, ADL, CES-D-9 and log-CRP burden; at least three baseline components required.	0.030 (0.006, 0.054)	0.013	0.014

Note. Sensitivity analyses use the primary covariates unless otherwise stated. No score includes cardiometabolic stage. Five-component baseline burden averages standardized worse cognition, worse grip, ADL, CES-D-9 and log-CRP, requiring at least three; non-depressive baseline burden omits CES-D-9 and requires at least three of four. The primary model retains its aligned baseline score. FDR correction covers all 19 displayed analyses.

Supplementary Table S7. Correlates of loneliness remission among women lonely at baseline.

Candidate remission correlate	Participants, n	Remitted participants, n	Exposed participants, n	Adjusted OR (95% CI)	P value
Partnered by 2015	1,064	477	733	2.16 (1.53, 3.05)	<0.001
Future child support available by 2015	997	450	555	1.65 (1.27, 2.15)	<0.001
Weekly contact with children by 2015	1,058	474	945	1.46 (0.96, 2.21)	0.076
New social activity by 2015	1,062	476	208	1.32 (0.93, 1.85)	0.118
Financial transfer from child by 2015	1,058	474	885	1.18 (0.83, 1.66)	0.355
Co-residence with child by 2015	1,058	474	502	1.08 (0.84, 1.39)	0.552
Child living nearby by 2015	1,042	468	890	1.13 (0.78, 1.62)	0.526
Any social activity by 2015	1,063	477	469	1.11 (0.86, 1.43)	0.422

Note. Restricted to women with high loneliness at baseline and complete trajectories, not necessarily the final primary outcome sample. Models adjust for age, education, rural residence, BMI, chronic-condition count, social isolation and baseline burden from cognition, grip, ADL, CES-D-9 and log-CRP; at least three burden components are required. Cardiometabolic stage is excluded. Odds ratios above 1 indicate higher odds of remission by 2015.

Supplementary Table S8. Baseline profile of remitted and non-remitted women with high loneliness at baseline.

Baseline characteristic	Remitted group, n	Remitted group, mean	Non-remitted group, n	Non-remitted group, mean	Standardized mean difference
Age	421	57.67	496	58.80	-0.131
Rural residence	421	0.72	496	0.74	-0.055
Baseline loneliness intensity	421	2.36	496	2.56	-0.413
Baseline CES-D-9 without loneliness item	421	12.79	496	15.38	-0.434
Five-component baseline burden	417	0.36	492	0.57	-0.362
Chronic disease count	421	1.24	496	1.49	-0.199
Social isolation index	421	0.78	496	0.87	-0.141
Baseline major disease	421	0.18	496	0.22	-0.102

Note. Standardized mean differences compare remitted and non-remitted women with high loneliness at baseline. Five-component baseline burden averages standardized worse cognition, worse grip, ADL, CES-D-9 and log-CRP, requiring at least three components; cardiometabolic stage is excluded.

Supplementary Table S9. Observation-interval burden contrast among women with high loneliness at baseline.

Analysis sample	Contrast	Participants, n	Adjusted beta (95% CI)	P value	Model note
Among women lonely at baseline	Non-remitted vs remitted after baseline loneliness	917	0.209 (0.127, 0.291)	<0.001	Adjusted for aligned baseline burden, baseline loneliness intensity, and baseline CES-D-9 where available.

Note. Compares women whose high baseline loneliness remitted with those who did not meet the remission definition. Remission required not-high loneliness at both subsequent assessments.

Supplementary Table S10. Subgroup analyses for two-year-scaled high-loneliness exposure.

Subgroup variable	Stratum	Participants, n	Adjusted beta (95% CI)	P value	FDR-adjusted P value
Residence	Urban	1,778	0.088 (0.054, 0.121)	<0.001	<0.001
Residence	Rural	3,126	0.058 (0.034, 0.081)	<0.001	<0.001
Age group	Age 45–59	3,158	0.066 (0.040, 0.091)	<0.001	<0.001
Age group	Age >=60	1,746	0.067 (0.037, 0.096)	<0.001	<0.001
Education	Higher education	2,086	0.076 (0.047, 0.106)	<0.001	<0.001
Education	Low education	2,818	0.060 (0.035, 0.085)	<0.001	<0.001
Baseline diabetes	No baseline diabetes	4,558	0.073 (0.053, 0.092)	<0.001	<0.001
Baseline diabetes	Baseline diabetes	300	-0.031 (-0.122, 0.060)	0.501	0.501

Note. Subgroup estimates use the two-year-scaled assigned high-loneliness exposure and the 2011–2018 observation-interval burden outcome; one unit approximates two assigned high-loneliness years.

Supplementary Table S11. Data-driven continuous loneliness-trajectory sensitivity analysis.

Data-driven loneliness trajectory	Participants, n	Mean loneliness score in 2011	Mean loneliness score in 2013	Mean loneliness score in 2015	Adjusted beta (95% CI)	P value
Data-driven low	3,207	1.13	1.15	1.09	Reference	
Data-driven persistent higher	597	2.48	3.61	2.52	0.189 (0.133, 0.245)	<0.001
Data-driven decreasing	569	3.46	1.28	1.66	-0.052 (-0.108, 0.004)	0.069
Data-driven increasing	531	1.42	1.31	3.51	0.155 (0.100, 0.210)	<0.001

Note. Data-driven sensitivity analysis based on continuous loneliness item scores.

Supplementary Table S12. HRS harmonized longitudinal trajectory comparison.

HRS loneliness trajectory	Participants, n	Adjusted beta (95% CI)	P value
Never high loneliness	1,186	0.000 (0.000, 0.000)	-
Remitted loneliness	150	-0.057 (-0.134, 0.020)	0.145
Incident loneliness	322	0.130 (0.061, 0.200)	<0.001
Intermittent loneliness	168	0.077 (-0.012, 0.167)	0.091
Persistent loneliness	199	0.155 (0.054, 0.255)	0.003

Note. The primary comparison included women in HRS with loneliness assessed at waves 8, 10, and 12. The CV-eligible sample comprised 2,080 women; complete-case regression samples were smaller. The final follow-up composite contained depressive symptoms, ADL limitations, and cognition, with no available grip-change component. HRS is a longitudinal comparison, not external validation. See Supplementary Tables S27 and S29 for definitions.

Supplementary Table S13. HRS domain-specific outcome comparison.

Outcome	Participants, n	Adjusted beta (95% CI)	P value
Composite burden worsening	2,025	0.055 (0.029, 0.081)	<0.001
Depressive symptoms worsening	2,026	0.192 (0.147, 0.236)	<0.001
ADL limitations worsening	2,079	0.064 (0.024, 0.103)	0.002
Cognitive decline	2,026	0.044 (0.003, 0.085)	0.033

Note. HRS domain construction is related to, but not identical with, CHARLS. The CES-D-8 outcome includes a loneliness item; global cognition is a supplied harmonized score. No wave-13 grip observation was available in the final primary sample, so grip change did not contribute to its composite. See Supplementary Table S29.

Supplementary Table S14. UK Biobank baseline phenotype convergence.

Outcome	Exposure	Participants, n	Adjusted beta (95% CI)	P value
Baseline psychosomatic burden	Joint loneliness indicator	182,571	0.394 (0.379, 0.409)	<0.001
Baseline psychosomatic burden	Social isolation	182,571	0.041 (0.037, 0.044)	<0.001
Depressive symptom score	Joint loneliness indicator	174,398	1.114 (1.078, 1.150)	<0.001
Depressive symptom score	Social isolation	174,398	0.102 (0.095, 0.109)	<0.001
Grip weakness	Joint loneliness indicator	182,228	0.071 (0.058, 0.084)	<0.001
Grip weakness	Social isolation	182,228	0.008 (0.005, 0.012)	<0.001
Inflammation burden	Joint loneliness indicator	172,134	0.027 (0.006, 0.048)	0.012
Inflammation burden	Social isolation	172,134	0.010 (0.004, 0.016)	<0.001

Note. Analyses were restricted to women aged ≥45 years at baseline. The exposure labelled loneliness is a joint indicator requiring both often feeling lonely and never/almost never being able to confide. It is not equivalent to the CHARLS item or HRS scale. All outcomes were measured at baseline, and the composite averaged depressive symptoms, grip weakness, and log-CRP where at least two were available. These results do not replicate trajectories or temporal associations. See Supplementary Table S30 for exact exposure coding and covariates.

Supplementary Table S15. CHARLS disease-specific confounding adjustment.

Analysis	Outcome	Exposure or contrast	Participants, n	Group n	Events, n	Scale	Estimate (95% CI)	P value	FDR-adjusted P value
D1 primary trajectory: chronic-count adjustment	Observation-interval burden	Never high	4904	3,108	-	beta	0.000 (0.000, 0.000)	-	-
D1 primary trajectory: chronic-count adjustment	Observation-interval burden	Remitted high	4904	421	-	beta	-0.083 (-0.144, -0.021)	0.009	0.012
D1 primary trajectory: chronic-count adjustment	Observation-interval burden	Incident high	4904	879	-	beta	0.191 (0.146, 0.236)	<0.001	<0.001
D1 primary trajectory: chronic-count adjustment	Observation-interval burden	Intermittent high	4904	321	-	beta	0.043 (-0.031, 0.118)	0.255	0.255
D1 primary trajectory: chronic-count adjustment	Observation-interval burden	Persistent high	4904	175	-	beta	0.178 (0.079, 0.276)	<0.001	<0.001
D2 primary trajectory: disease-specific adjustment	Observation-interval burden	Never high	4740	3,011	-	beta	0.000 (0.000, 0.000)	-	-
D2 primary trajectory: disease-specific adjustment	Observation-interval burden	Remitted high	4740	413	-	beta	-0.080 (-0.143, -0.017)	0.013	0.017
D2 primary trajectory: disease-specific adjustment	Observation-interval burden	Incident high	4740	837	-	beta	0.190 (0.144, 0.236)	<0.001	<0.001
D2 primary trajectory: disease-specific adjustment	Observation-interval burden	Intermittent high	4740	311	-	beta	0.038 (-0.038, 0.114)	0.331	0.331
D2 primary trajectory: disease-specific adjustment	Observation-interval burden	Persistent high	4740	168	-	beta	0.167 (0.066, 0.269)	0.001	0.002
D3 primary two-year-scaled exposure: chronic-count adjustment	Observation-interval burden	Two-year-scaled high-loneliness exposure	4904	-	-	beta	0.066 (0.047, 0.085)	<0.001	<0.001
D4 primary two-year-scaled exposure: disease-specific adjustment	Observation-interval burden	Two-year-scaled high-loneliness exposure	4740	-	-	beta	0.063 (0.044, 0.083)	<0.001	<0.001
D5 post-2015 two-year-scaled exposure: chronic-count adjustment	2015–2018 worsening score	Two-year-scaled high-loneliness exposure	4900	-	-	beta	-0.065 (-0.089, -0.041)	<0.001	<0.001

Analysis	Outcome	Exposure or contrast	Participants, n	Group n	Events, n	Scale	Estimate (95% CI)	P value	FDR-adjusted P value
D6 post-2015 two-year-scaled exposure: disease-specific adjustment	2015–2018 worsening score	Two-year-scaled high-loneliness exposure	4736	-	-	beta	-0.063 (-0.089, -0.038)	<0.001	<0.001

Note. Disease-specific models replaced chronic disease count with individual baseline disease indicators.

Supplementary Table S16. Secondary endpoint-eligible CHARLS 2015–2018 incident-outcome models.

Analysis	Outcome	Exposure or contrast	Participants, n	Group n	Events, n	Scale	Estimate (95% CI)	P value	FDR-adjusted P value
cumulative	Incident ADL limitation	Two high-loneliness person-years	4,394	-	530	RR	1.186 (1.101, 1.278)	<0.001	<0.001
trajectory	Incident ADL limitation	Never high	4,394	2,920	530	RR	1.000 (1.000, 1.000)	-	-
trajectory	Incident ADL limitation	Remitted high	4,394	370	530	RR	1.047 (0.787, 1.394)	0.751	0.751
trajectory	Incident ADL limitation	Incident high	4,394	733	530	RR	1.254 (1.023, 1.537)	0.029	0.043
trajectory	Incident ADL limitation	Intermittent high	4,394	251	530	RR	1.740 (1.346, 2.250)	<0.001	<0.001
trajectory	Incident ADL limitation	Persistent high	4,394	120	530	RR	1.582 (1.125, 2.226)	0.008	0.016
cumulative	Incident mobility limitation	Two high-loneliness person-years	1,611	-	772	RR	1.089 (1.031, 1.151)	0.002	0.003
trajectory	Incident mobility limitation	Never high	1,611	1,211	772	RR	1.000 (1.000, 1.000)	-	-
trajectory	Incident mobility limitation	Remitted high	1,611	119	772	RR	1.048 (0.875, 1.255)	0.609	0.656
trajectory	Incident mobility limitation	Incident high	1,611	200	772	RR	1.134 (0.986, 1.305)	0.078	0.109
trajectory	Incident mobility limitation	Intermittent high	1,611	59	772	RR	1.331 (1.080, 1.641)	0.007	0.015
trajectory	Incident mobility limitation	Persistent high	1,611	22	772	RR	1.291 (0.966, 1.726)	0.084	0.112
cumulative	Incident lower-body mobility limitation	Two high-loneliness person-years	1,736	-	801	RR	1.080 (1.022, 1.141)	0.006	0.006
trajectory	Incident lower-body mobility limitation	Never high	1,736	1,289	801	RR	1.000 (1.000, 1.000)	-	-
trajectory	Incident lower-body mobility limitation	Remitted high	1,736	134	801	RR	1.030 (0.866, 1.226)	0.736	0.751
trajectory	Incident lower-body mobility limitation	Incident high	1,736	223	801	RR	1.053 (0.912, 1.217)	0.483	0.541

Analysis	Outcome	Exposure or contrast	Participants, n	Group n	Events, n	Scale	Estimate (95% CI)	P value	FDR-adjusted P value
trajectory	Incident lower-body mobility limitation	Intermittent high	1,736	64	801	RR	1.314 (1.067, 1.619)	0.010	0.018
trajectory	Incident lower-body mobility limitation	Persistent high	1,736	26	801	RR	1.434 (1.126, 1.827)	0.004	0.009
cumulative	Incident 100 m walking difficulty	Two high-loneliness person-years	4,718	-	418	RR	1.138 (1.044, 1.241)	0.003	0.004
trajectory	Incident 100 m walking difficulty	Never high	4,718	3,057	418	RR	1.000 (1.000, 1.000)	-	-
trajectory	Incident 100 m walking difficulty	Remitted high	4,718	399	418	RR	1.265 (0.942, 1.699)	0.118	0.150
trajectory	Incident 100 m walking difficulty	Incident high	4,718	827	418	RR	1.109 (0.871, 1.411)	0.401	0.468
trajectory	Incident 100 m walking difficulty	Intermittent high	4,718	285	418	RR	1.442 (1.047, 1.987)	0.025	0.039
trajectory	Incident 100 m walking difficulty	Persistent high	4,718	150	418	RR	1.739 (1.210, 2.500)	0.003	0.008
cumulative	Incident chair-stand difficulty	Two high-loneliness person-years	3,247	-	862	RR	1.186 (1.123, 1.253)	<0.001	<0.001
trajectory	Incident chair-stand difficulty	Never high	3,247	2,246	862	RR	1.000 (1.000, 1.000)	-	-
trajectory	Incident chair-stand difficulty	Remitted high	3,247	275	862	RR	1.392 (1.166, 1.661)	<0.001	0.001
trajectory	Incident chair-stand difficulty	Incident high	3,247	498	862	RR	1.285 (1.108, 1.489)	<0.001	0.003
trajectory	Incident chair-stand difficulty	Intermittent high	3,247	153	862	RR	1.650 (1.333, 2.043)	<0.001	<0.001
trajectory	Incident chair-stand difficulty	Persistent high	3,247	75	862	RR	1.719 (1.319, 2.241)	<0.001	<0.001
cumulative	Incident elevated CES-D-9 symptoms (>=10)	Two high-loneliness person-years	2,942	-	846	RR	1.261 (1.184, 1.343)	<0.001	<0.001
trajectory	Incident elevated CES-D-9 symptoms (>=10)	Never high	2,942	2,280	846	RR	1.000 (1.000, 1.000)	-	-
trajectory	Incident elevated CES-D-9 symptoms (>=10)	Remitted high	2,942	237	846	RR	1.306 (1.083, 1.576)	0.005	0.011
trajectory	Incident elevated CES-D-9 symptoms (>=10)	Incident high	2,942	286	846	RR	1.520 (1.296, 1.783)	<0.001	<0.001
trajectory	Incident elevated CES-D-9 symptoms (>=10)	Intermittent high	2,942	112	846	RR	1.774 (1.444, 2.180)	<0.001	<0.001

Analysis	Outcome	Exposure or contrast	Participants, n	Group n	Events, n	Scale	Estimate (95% CI)	P value	FDR-adjusted P value
trajectory	Incident elevated CES-D-9 symptoms (>=10)	Persistent high	2,942	27	846	RR	1.743 (1.197, 2.540)	0.004	0.009
cumulative	Incident heart disease or stroke	Two high-loneliness person-years	3,919	-	532	RR	1.211 (1.122, 1.307)	<0.001	<0.001
trajectory	Incident heart disease or stroke	Never high	3,919	2,578	532	RR	1.000 (1.000, 1.000)	-	-
trajectory	Incident heart disease or stroke	Remitted high	3,919	324	532	RR	1.190 (0.891, 1.591)	0.239	0.291
trajectory	Incident heart disease or stroke	Incident high	3,919	676	532	RR	1.369 (1.117, 1.677)	0.002	0.008
trajectory	Incident heart disease or stroke	Intermittent high	3,919	223	532	RR	1.462 (1.084, 1.971)	0.013	0.021
trajectory	Incident heart disease or stroke	Persistent high	3,919	118	532	RR	1.900 (1.346, 2.683)	<0.001	0.001

Note. These secondary models used endpoint-specific eligibility from the full trajectory-eligible CHARLS cohort, modified Poisson relative risks, and separate multiplicity families: seven cumulative-exposure endpoint tests and 28 nonreference trajectory contrasts.

Supplementary Table S17. CHARLS 2015–2018 continuous change-outcome models.

Analysis	Outcome	Exposure or contrast	Participants, n	Group n	Events, n	Scale	Estimate (95% CI)	P value	FDR-adjusted P value
A1 trajectory: 2015–2018 change, baseline confounders	2015–2018 worsening score	Never high	4900	3,106	-	beta	0.000 (0.000, 0.000)	-	-
A1 trajectory: 2015–2018 change, baseline confounders	2015–2018 worsening score	Remitted high	4900	420	-	beta	0.033 (-0.044, 0.109)	0.403	0.403
A1 trajectory: 2015–2018 change, baseline confounders	2015–2018 worsening score	Incident high	4900	879	-	beta	-0.150 (-0.206, -0.095)	<0.001	<0.001
A1 trajectory: 2015–2018 change, baseline confounders	2015–2018 worsening score	Intermittent high	4900	321	-	beta	-0.102 (-0.208, 0.003)	0.057	0.076
A1 trajectory: 2015–2018 change, baseline confounders	2015–2018 worsening score	Persistent high	4900	174	-	beta	-0.196 (-0.332, -0.060)	0.005	0.010
A2 trajectory: 2015–2018 change plus 2015 burden	2015–2018 worsening score	Never high	4857	3,080	-	beta	0.000 (0.000, 0.000)	-	-
A2 trajectory: 2015–2018 change plus 2015 burden	2015–2018 worsening score	Remitted high	4857	416	-	beta	0.114 (0.039, 0.188)	0.003	0.011

Analysis	Outcome	Exposure or contrast	Participants, n	Group n	Events, n	Scale	Estimate (95% CI)	P value	FDR-adjusted P value
A2 trajectory: 2015–2018 change plus 2015 burden	2015–2018 worsening score	Incident high	4857	870	-	beta	0.026 (-0.026, 0.079)	0.331	0.331
A2 trajectory: 2015–2018 change plus 2015 burden	2015–2018 worsening score	Intermittent high	4857	317	-	beta	0.084 (-0.011, 0.179)	0.082	0.109
A2 trajectory: 2015–2018 change plus 2015 burden	2015–2018 worsening score	Persistent high	4857	174	-	beta	0.112 (-0.013, 0.238)	0.080	0.109
A3 two-year-scaled exposure: 2015–2018 change, baseline confounders	2015–2018 worsening score	Two-year-scaled high-loneliness exposure	4900	-	-	beta	-0.065 (-0.089, -0.041)	<0.001	<0.001
A4 two-year-scaled exposure: 2015–2018 change plus 2015 burden	2015–2018 worsening score	Two-year-scaled high-loneliness exposure	4857	-	-	beta	0.030 (0.006, 0.054)	0.013	0.013

Note. The 2015–2018 change outcome averages standardized deterioration in cognition, four-item ADL limitations and CES-D-9 symptoms, requiring at least two domains. Additional 2015-burden adjustment averages standardized worse cognition, worse grip, ADL, CES-D-9 and log-CRP, requiring at least three of five components and excluding cardiometabolic stage. The primary composite is separate and includes physical measurements ending in either 2015 or 2018. FDR correction is applied within each analysis specification.

Supplementary Table S18. HRS wave 12–13 post-exposure change models.

Analysis	Outcome	Exposure or contrast	Participants, n	Group n	Events, n	Scale	Estimate (95% CI)	P value	FDR-adjusted P value
C1 HRS wave 12–13 change: trajectory, baseline confounders	HRS wave 12–13 worsening z	Never high	2,022	1,185	-	beta	0.000 (0.000, 0.000)	-	-
C1 HRS wave 12–13 change: trajectory, baseline confounders	HRS wave 12–13 worsening z	Remitted high	2,022	151	-	beta	-0.006 (-0.103, 0.091)	0.903	0.903
C1 HRS wave 12–13 change: trajectory, baseline confounders	HRS wave 12–13 worsening z	Incident high	2,022	321	-	beta	-0.026 (-0.103, 0.051)	0.513	0.798
C1 HRS wave 12–13 change: trajectory, baseline confounders	HRS wave 12–13 worsening z	Intermittent high	2,022	166	-	beta	-0.024 (-0.114, 0.066)	0.599	0.798
C1 HRS wave 12–13 change: trajectory, baseline confounders	HRS wave 12–13 worsening z	Persistent high	2,022	199	-	beta	-0.069 (-0.174, 0.035)	0.193	0.771
C2 HRS wave 12–13 change plus wave 12 burden: trajectory	HRS wave 12–13 worsening z	Never high	2,022	1,185	-	beta	0.000 (0.000, 0.000)	-	-
C2 HRS wave 12–13 change plus wave 12 burden: trajectory	HRS wave 12–13 worsening z	Remitted high	2,022	151	-	beta	0.020 (-0.073, 0.113)	0.676	0.676

Analysis	Outcome	Exposure or contrast	Participants, n	Group n	Events, n	Scale	Estimate (95% CI)	P value	FDR-adjusted P value
C2 HRS wave 12–13 change plus wave 12 burden: trajectory	HRS wave 12–13 worsening z	Incident high	2,022	321	-	beta	0.043 (-0.030, 0.117)	0.248	0.330
C2 HRS wave 12–13 change plus wave 12 burden: trajectory	HRS wave 12–13 worsening z	Intermittent high	2,022	166	-	beta	0.067 (-0.019, 0.154)	0.126	0.286
C2 HRS wave 12–13 change plus wave 12 burden: trajectory	HRS wave 12–13 worsening z	Persistent high	2,022	199	-	beta	0.076 (-0.026, 0.177)	0.143	0.286
C3 HRS wave 12–13 change: repeated high-loneliness waves, baseline confounders	HRS wave 12–13 worsening z	Per additional high-loneliness assessment	2,022	-	-	beta	-0.016 (-0.043, 0.011)	0.249	0.249
C4 HRS wave 12–13 change plus wave 12 burden: repeated high-loneliness waves	HRS wave 12–13 worsening z	Per additional high-loneliness assessment	2,022	-	-	beta	0.031 (0.004, 0.057)	0.022	0.022

Note. HRS wave 12–13 models assess change after the final loneliness measurement. The actual follow-up components were depressive symptoms, ADL limitations, and cognition; no grip-change component was available. The primary and stricter time-separated models have different outcome intervals.

Supplementary Table S19. HRS disease-specific confounding adjustment.

Analysis	Outcome	Exposure or contrast	Participants, n	Events, n	Scale	Estimate (95% CI)	P value	FDR-adjusted P value
D7 HRS original repeated-wave exposure: chronic-count adjustment	Composite worsening	Per additional high-loneliness assessment	2,025	-	beta	0.055 (0.029, 0.081)	<0.001	<0.001
D8 HRS original repeated-wave exposure: disease-specific adjustment	Composite worsening	Per additional high-loneliness assessment	2,025	-	beta	0.054 (0.028, 0.080)	<0.001	<0.001
D9 HRS wave 12–13 repeated-wave exposure: chronic-count adjustment	Wave 12–13 composite worsening	Per additional high-loneliness assessment	2,022	-	beta	-0.016 (-0.043, 0.011)	0.249	0.249
D10 HRS wave 12–13 repeated-wave exposure: disease-specific adjustment	Wave 12–13 composite worsening	Per additional high-loneliness assessment	2,022	-	beta	-0.016 (-0.043, 0.011)	0.237	0.237

Note. HRS disease-specific models replaced chronic disease count with hypertension, diabetes, heart disease, and stroke indicators.

Supplementary Table S20. Unadjusted CHARLS trajectory and domain models.

Analysis	Domain	Outcome	Exposure or contrast	Participants, n	Group n	Events, n	Scale	Estimate (95% CI)	P value	FDR-adjusted P value
F1 unadjusted Table 2-style trajectory	-	Primary composite observation-interval burden	Never high	4904	3,108	-	beta	0.000 (0.000, 0.000)	-	-
F1 unadjusted Table 2-style trajectory	-	Primary composite observation-interval burden	Remitted high	4904	421	-	beta	-0.227 (-0.293, -0.160)	<0.001	<0.001
F1 unadjusted Table 2-style trajectory	-	Primary composite observation-interval burden	Incident high	4904	879	-	beta	0.139 (0.091, 0.187)	<0.001	<0.001
F1 unadjusted Table 2-style trajectory	-	Primary composite observation-interval burden	Intermittent high	4904	321	-	beta	-0.174 (-0.251, -0.097)	<0.001	<0.001
F1 unadjusted Table 2-style trajectory	-	Primary composite observation-interval burden	Persistent high	4904	175	-	beta	-0.097 (-0.201, 0.008)	0.069	0.069
F2 unadjusted Table 3-style two-year-scaled exposure	Overall	Primary composite observation-interval burden	Two-year-scaled high-loneliness exposure	4904	-	-	beta	-0.009 (-0.029, 0.011)	0.370	0.535
F2 unadjusted Table 3-style two-year-scaled exposure	Cognitive	Cognitive score decline	Two-year-scaled high-loneliness exposure	3701	-	-	beta	0.045 (0.010, 0.081)	0.012	0.027
F2 unadjusted Table 3-style two-year-scaled exposure	Physical	Physical function composite	Two-year-scaled high-loneliness exposure	4903	-	-	beta	0.030 (0.010, 0.050)	0.003	0.009
F2 unadjusted Table 3-style two-year-scaled exposure	Physical	Grip strength decline	Two-year-scaled high-loneliness exposure	3684	-	-	beta	0.007 (-0.026, 0.040)	0.674	0.674
F2 unadjusted Table 3-style two-year-scaled exposure	Physical	Mobility limitation worsening	Two-year-scaled high-loneliness exposure	4903	-	-	beta	0.056 (0.024, 0.089)	<0.001	0.004
F2 unadjusted Table 3-style two-year-scaled exposure	Physical	Walking-test time increase	Two-year-scaled high-loneliness exposure	1374	-	-	beta	0.020 (-0.025, 0.065)	0.389	0.535
F2 unadjusted Table 3-style two-year-scaled exposure	Physical	Chair-stand slowing	Two-year-scaled high-loneliness exposure	3652	-	-	beta	0.009 (-0.026, 0.044)	0.615	0.674
F2 unadjusted Table 3-style two-year-scaled exposure	Physical	ADL limitation worsening	Two-year-scaled high-loneliness exposure	4855	-	-	beta	0.052 (0.019, 0.086)	0.002	0.009
F2 unadjusted Table 3-style two-year-scaled exposure	Metabolic	Metabolic biomarker deterioration	Two-year-scaled high-loneliness exposure	3026	-	-	beta	-0.005 (-0.021, 0.011)	0.521	0.637

Analysis	Domain	Outcome	Exposure or contrast	Participants, n	Group n	Events, n	Scale	Estimate (95% CI)	P value	FDR-adjusted P value
F2 unadjusted Table 3-style two-year-scaled exposure	Inflammatory	Inflammation increase	Two-year-scaled high-loneliness exposure	3026	-	-	beta	0.030 (-0.005, 0.065)	0.091	0.167
F2 unadjusted Table 3-style two-year-scaled exposure	Psychological	Depressive symptoms worsening	Two-year-scaled high-loneliness exposure	4851	-	-	beta	-0.084 (-0.117, -0.051)	<0.001	<0.001

Note. Unadjusted models omit baseline covariates and baseline outcome adjustment. Cardiometabolic-stage-derived endpoints were excluded. FDR correction is separate for the four nonreference trajectory contrasts and the 11 cumulative-exposure domain tests.

Supplementary Table S21. Unadjusted HRS comparison models.

Analysis	Outcome	Exposure or contrast	Participants, n	Group n	Events, n	Scale	Estimate (95% CI)	P value	FDR-adjusted P value
F3 HRS unadjusted original trajectory	Composite worsening	Never high	2,080	1,214	-	beta	0.000 (0.000, 0.000)	-	-
F3 HRS unadjusted original trajectory	Composite worsening	Remitted high	2,080	153	-	beta	-0.137 (-0.221, -0.054)	0.001	0.005
F3 HRS unadjusted original trajectory	Composite worsening	Incident high	2,080	336	-	beta	0.087 (0.012, 0.162)	0.022	0.045
F3 HRS unadjusted original trajectory	Composite worsening	Intermittent high	2,080	171	-	beta	-0.072 (-0.172, 0.027)	0.155	0.207
F3 HRS unadjusted original trajectory	Composite worsening	Persistent high	2,080	206	-	beta	-0.013 (-0.121, 0.095)	0.812	0.812
F4 HRS unadjusted wave 12–13 trajectory	Wave 12–13 composite worsening	Never high	2,078	1,214	-	beta	0.000 (0.000, 0.000)	-	-
F4 HRS unadjusted wave 12–13 trajectory	Wave 12–13 composite worsening	Remitted high	2,078	154	-	beta	-0.016 (-0.111, 0.079)	0.742	0.742
F4 HRS unadjusted wave 12–13 trajectory	Wave 12–13 composite worsening	Incident high	2,078	335	-	beta	-0.019 (-0.095, 0.058)	0.631	0.742
F4 HRS unadjusted wave 12–13 trajectory	Wave 12–13 composite worsening	Intermittent high	2,078	169	-	beta	-0.029 (-0.119, 0.061)	0.523	0.742
F4 HRS unadjusted wave 12–13 trajectory	Wave 12–13 composite worsening	Persistent high	2,078	206	-	beta	-0.065 (-0.167, 0.037)	0.210	0.742
F5 HRS unadjusted original repeated-wave exposure	Composite worsening	Per additional high-loneliness assessment	2,080	-	-	beta	-0.003 (-0.030, 0.025)	0.851	0.851
F6 HRS unadjusted wave 12–13 repeated-wave exposure	Wave 12–13 composite worsening	Per additional high-loneliness assessment	2,078	-	-	beta	-0.016 (-0.042, 0.011)	0.248	0.248

Note. Unadjusted HRS comparison models omit baseline covariates and baseline outcome adjustment.

Supplementary Table S22. Early attrition and trajectory-completion analyses.

Panel A. Study flow for early attrition.

Flow step	Participants, n
Baseline-eligible women aged >=45 with 2011 loneliness	7980
Completed loneliness measures in 2011, 2013, and 2015	5677
Included in final primary analysis with an observed primary composite	4904

Panel B. Baseline profile by 2011–2015 loneliness-measure completion.

Baseline characteristic	Complete n	Complete mean/proportion	Incomplete n	Incomplete mean/proportion	Standardized difference
Age	5677	57.649	2303	61.138	-0.343
Rural residence	5677	0.63	2303	0.481	0.303
Low education	5677	0.588	2303	0.581	0.013
High education	5677	0.073	2303	0.135	-0.205
Body mass index	5677	23.949	2303	23.422	0.145
Ever smoking	5677	0.076	2303	0.095	-0.069
Drank last year	5677	0.119	2303	0.112	0.024
Baseline high loneliness	5677	0.19	2303	0.221	-0.077
Chronic disease count	5677	1.106	2303	1.174	-0.058
Social isolation index	5677	0.667	2303	0.76	-0.148
Aligned baseline burden	5672	0.19	2303	0.272	-0.116
Baseline CES-D-9 without loneliness item	5677	8.742	2303	8.743	-0

Panel C. Logistic model for completion of 2011–2015 loneliness measures.

Predictor	Participants, n	Completed, n	Adjusted OR (95% CI)	P value	FDR-adjusted P value
Low versus high education	7975	5672	2.175 (1.807, 2.617)	<0.001	<0.001
Middle versus high education	7975	5672	2.162 (1.805, 2.591)	<0.001	<0.001
Baseline high loneliness	7975	5672	0.896 (0.785, 1.022)	0.102	0.141
Age in years	7975	5672	0.966 (0.960, 0.972)	<0.001	<0.001
Rural residence	7975	5672	1.799 (1.616, 2.001)	<0.001	<0.001
Body mass index	7975	5672	1.033 (1.019, 1.048)	<0.001	<0.001
Ever smoking	7975	5672	0.897 (0.751, 1.072)	0.232	0.256
Alcohol use in previous year	7975	5672	1.103 (0.941, 1.292)	0.228	0.256
Chronic-condition count	7975	5672	1.013 (0.968, 1.061)	0.574	0.574
Social-isolation score	7975	5672	0.928 (0.853, 1.010)	0.084	0.133
Aligned baseline burden	7975	5672	0.881 (0.807, 0.962)	0.005	0.009

Note. Attrition analyses describe completion of the 2011–2015 loneliness trajectory window before the 2018 outcome wave.

Supplementary Table S23. Fixed paired cross-validation performance for single-wave and repeated-loneliness models.

Analysis	Cohort	Participants, n	Metric	Single-wave reference model	Repeated-measure model	Difference (95% CI)	Preferred direction
Primary	CHARLS	4,904	R^2	0.1765 (0.1551, 0.1960)	0.1943 (0.1719, 0.2144)	0.0178 (0.0102, 0.0251)	higher is better
Primary	CHARLS	4,904	RMSE	0.5625 (0.5504, 0.5747)	0.5564 (0.5445, 0.5687)	-0.0061 (-0.0087, -0.0035)	lower is better
Primary	CHARLS	4,904	MAE	0.4439 (0.4343, 0.4537)	0.4382 (0.4287, 0.4479)	-0.0057 (-0.0080, -0.0033)	lower is better
Primary	CHARLS	4,904	Calibration intercept	0.0001 (-0.0153, 0.0163)	0.0001 (-0.0158, 0.0165)	-0.0001 (-0.0025, 0.0023)	ideal 0
Primary	CHARLS	4,904	Calibration slope	0.9880 (0.9224, 1.0484)	0.9870 (0.9239, 1.0463)	-0.0010 (-0.0218, 0.0206)	ideal 1
Primary	HRS	2,080	R^2	0.1765 (0.1384, 0.2135)	0.1911 (0.1519, 0.2293)	0.0146 (0.0043, 0.0253)	higher is better
Primary	HRS	2,080	RMSE	0.5017 (0.4779, 0.5246)	0.4972 (0.4739, 0.5199)	-0.0045 (-0.0078, -0.0013)	lower is better
Primary	HRS	2,080	MAE	0.3674 (0.3527, 0.3815)	0.3640 (0.3492, 0.3782)	-0.0034 (-0.0063, -0.0007)	lower is better
Primary	HRS	2,080	Calibration intercept	-0.0019 (-0.0236, 0.0213)	-0.0020 (-0.0239, 0.0214)	-0.0000 (-0.0038, 0.0037)	ideal 0
Primary	HRS	2,080	Calibration slope	0.9734 (0.8634, 1.0847)	0.9723 (0.8691, 1.0780)	-0.0012 (-0.0315, 0.0304)	ideal 1
Incident ADL at landmark	CHARLS	4,394	AUC	0.7065 (0.6838, 0.7296)	0.7089 (0.6864, 0.7320)	0.0024 (-0.0019, 0.0066)	higher is better
Incident ADL at landmark	CHARLS	4,394	Brier score	0.0994 (0.0927, 0.1061)	0.0992 (0.0926, 0.1059)	-0.0002 (-0.0006, 0.0003)	lower is better
Incident ADL at landmark	CHARLS	4,394	Calibration intercept	-0.0846 (-0.3130, 0.1605)	-0.0951 (-0.3195, 0.1429)	-0.0106 (-0.0561, 0.0345)	ideal 0
Incident ADL at landmark	CHARLS	4,394	Calibration slope	0.9534 (0.8374, 1.0812)	0.9475 (0.8363, 1.0704)	-0.0059 (-0.0302, 0.0190)	ideal 1
Incident ADL at landmark	HRS	1,873	AUC	0.7072 (0.6638, 0.7494)	0.7079 (0.6657, 0.7494)	0.0007 (-0.0068, 0.0083)	higher is better
Incident ADL at landmark	HRS	1,873	Brier score	0.0753 (0.0661, 0.0851)	0.0756 (0.0663, 0.0852)	0.0002 (-0.0002, 0.0007)	lower is better
Incident ADL at landmark	HRS	1,873	Calibration intercept	-0.2152 (-0.6813, 0.2449)	-0.2556 (-0.7039, 0.1847)	-0.0403 (-0.1239, 0.0396)	ideal 0
Incident ADL at landmark	HRS	1,873	Calibration slope	0.8975 (0.6940, 1.1092)	0.8780 (0.6877, 1.0807)	-0.0195 (-0.0594, 0.0171)	ideal 1

Note. Reference and repeated-measure models used identical folds. In the primary CHARLS comparison, the reference uses 2011 loneliness and the repeated model adds 2013 and 2015; in HRS, the reference uses wave 8 and the repeated model adds waves 10 and 12. Secondary incident-ADL comparisons use the 2015 landmark alone versus all three assessments in CHARLS, and wave 12 alone versus waves 8, 10, and 12 in HRS. The primary decision statistic was the median across ten paired seeds: ΔR^2=0.0177 and RMSE change −1.083% in CHARLS, and ΔR^2=0.0145 and RMSE change −0.884% in HRS. The comparison criterion was fixed before paired models were run; averaged out-of-fold rows are complementary summaries. Predictor imputation and encoding were fitted within training folds, while outcome standardization remained fixed. Performance differences do not establish clinical utility or resolve overlapping measurement windows.

Supplementary Figure S1. Data-driven continuous loneliness-trajectory sensitivity analysis.

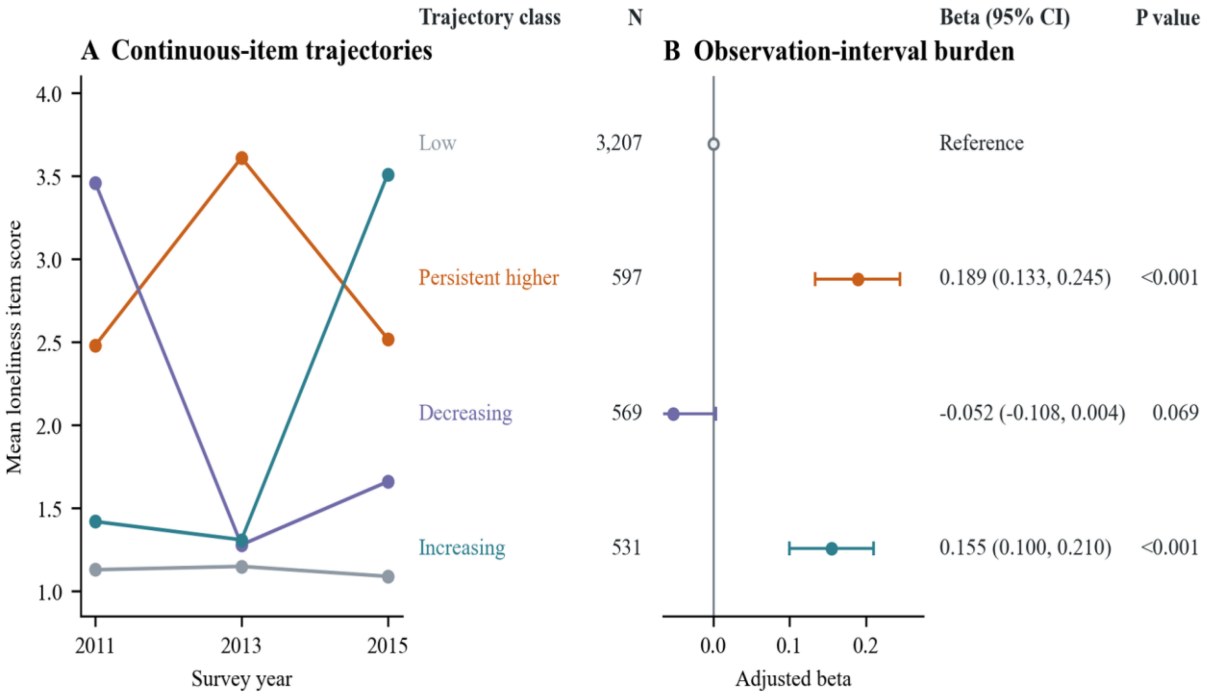

Note. This sensitivity analysis used data-driven grouping of continuous loneliness item scores and compared adjusted estimates for the 2011–2018 observation-interval burden.

Supplementary Table S24. Sex-stratified incremental-information sensitivity analyses.

Cohort	Sex	Participants, n	Median per-seed ΔR²	Averaged OOF ΔR² (95% CI)	Median relative RMSE change
CHARLS	Women	4,904	0.0177	0.0178 (0.0103, 0.0254)	-1.083%
CHARLS	Men	4,287	0.0146	0.0148 (0.0067, 0.0228)	-0.867%
HRS	Women	2,080	0.0145	0.0146 (0.0040, 0.0250)	-0.884%
HRS	Men	1,372	0.0031	0.0029 (-0.0072, 0.0124)	-0.183%

Note. OOF, out of fold; RMSE, root mean squared error. Each sex-specific analysis used the same fixed reference and repeated-measure models, ten seeds, paired five-fold assignments, training-fold preprocessing, and 2,000 participant-bootstrap resamples used elsewhere in the manuscript. These analyses were conducted as additional post hoc sensitivity analyses.

Supplementary Table S25. Sex- and age-stratified association sensitivity analyses.

Cohort/analysis	Contrast	Participants, n	Adjusted beta (95% CI)	P value	Interpretation
CHARLS	Women: cumulative loneliness	4,315	0.062 (0.041, 0.082)	<0.001	Sex-stratified cumulative-exposure association
CHARLS	Men: cumulative loneliness	3,757	0.063 (0.040, 0.087)	<0.001	Sex-stratified cumulative-exposure association
HRS	Women: cumulative loneliness	2,025	0.055 (0.029, 0.081)	<0.001	Sex-stratified cumulative-exposure association
HRS	Men: cumulative loneliness	1,359	0.054 (0.021, 0.088)	0.002	Sex-stratified cumulative-exposure association
CHARLS	Cumulative loneliness × female sex	8,072	-0.008 (-0.037, 0.021)	0.597	Sex interaction; null indicates no detected difference
HRS	Cumulative loneliness × female sex	3,384	-0.011 (-0.053, 0.030)	0.587	Sex interaction; null indicates no detected difference
women in CHARLS	Age 45–59	2,765	0.064 (0.037, 0.091)	<0.001	Age-stratified cumulative-exposure association
women in CHARLS	Age 60–74	1,438	0.056 (0.023, 0.089)	<0.001	Age-stratified cumulative-exposure association
women in CHARLS	Age ≥75	112	0.075 (-0.052, 0.201)	0.248	Age-stratified cumulative-exposure association
women in CHARLS	Omnibus cumulative exposure × age group	4,315	χ^2=0.998; df=2	0.607	Omnibus interaction

Note. CHARLS coefficients are per two-year-scaled duration-weighted exposure unit; HRS coefficients are per additional high-loneliness assessment. Sex interactions were estimated in pooled cohort-specific models. Age groups were 45–59, 60–74, and ≥75 years. These post hoc sensitivity analyses were not part of the prespecified primary analysis.

Supplementary Table S26. Equal-weight sensitivity analyses across outcome domains.

Outcome	Exposure scheme	Participants, n	Beta per 1-SD exposure (95% CI)	P value	FDR-adjusted P value
Primary observation-interval burden	Duration-weighted 2:2:3	4,904	0.064 (0.045, 0.083)	<0.001	<0.001
Primary observation-interval burden	Equal-weighted 1:1:1	4,904	0.057 (0.038, 0.076)	<0.001	<0.001
Cognitive score decline	Duration-weighted 2:2:3	3,701	0.045 (0.015, 0.075)	0.004	0.006
Cognitive score decline	Equal-weighted 1:1:1	3,701	0.042 (0.011, 0.072)	0.007	0.011
Physical function composite	Duration-weighted 2:2:3	4,860	0.054 (0.033, 0.074)	<0.001	<0.001
Physical function composite	Equal-weighted 1:1:1	4,860	0.053 (0.032, 0.073)	<0.001	<0.001
Grip strength decline	Duration-weighted 2:2:3	3,684	0.045 (0.020, 0.070)	<0.001	<0.001
Grip strength decline	Equal-weighted 1:1:1	3,684	0.044 (0.019, 0.069)	<0.001	0.001
Mobility limitation worsening	Duration-weighted 2:2:3	4,860	0.057 (0.022, 0.091)	0.001	0.002
Mobility limitation worsening	Equal-weighted 1:1:1	4,860	0.051 (0.016, 0.086)	0.004	0.007
Walking-test time increase	Duration-weighted 2:2:3	1,374	0.016 (-0.034, 0.065)	0.532	0.650
Walking-test time increase	Equal-weighted 1:1:1	1,374	0.020 (-0.032, 0.072)	0.452	0.539
Chair-stand slowing	Duration-weighted 2:2:3	3,652	0.000 (-0.035, 0.036)	0.988	0.988
Chair-stand slowing	Equal-weighted 1:1:1	3,652	-0.003 (-0.039, 0.033)	0.877	0.877
ADL limitation worsening	Duration-weighted 2:2:3	4,855	0.081 (0.051, 0.112)	<0.001	<0.001
ADL limitation worsening	Equal-weighted 1:1:1	4,855	0.084 (0.054, 0.114)	<0.001	<0.001
Metabolic biomarker deterioration	Duration-weighted 2:2:3	3,026	-0.004 (-0.020, 0.012)	0.619	0.681
Metabolic biomarker deterioration	Equal-weighted 1:1:1	3,026	-0.006 (-0.021, 0.010)	0.490	0.539
Inflammation increase	Duration-weighted 2:2:3	3,026	0.014 (-0.015, 0.042)	0.345	0.474
Inflammation increase	Equal-weighted 1:1:1	3,026	0.014 (-0.014, 0.042)	0.337	0.463
Depressive symptoms worsening	Duration-weighted 2:2:3	4,851	0.195 (0.166, 0.225)	<0.001	<0.001
Depressive symptoms worsening	Equal-weighted 1:1:1	4,851	0.187 (0.156, 0.217)	<0.001	<0.001

Note. Duration weighting used 2:2:3 for the 2011, 2013 and 2015 indicators; equal weighting used 1:1:1. Exposures were standardized within each outcome-specific complete-case sample. The two scores correlated at r=0.9897 in the primary sample of 4,904 women. Physical-function, mobility and metabolic models use five-component baseline burden without cardiometabolic stage; the primary model retains aligned baseline burden. FDR correction was applied separately within each weighting scheme across 11 outcomes.

Supplementary Table S27. Cohort roles and analytic sample characteristics.

Characteristic	CHARLS	HRS	UK Biobank
Setting and study role	China; primary longitudinal analysis of incremental information and domain-specific burden.	United States; longitudinal comparison using related, but non-equivalent, exposure and outcome measures.	United Kingdom; cross-sectional comparison of related psychological and physical phenotypes at baseline.
Primary sex restriction and age	Women aged ≥45 years in 2011.	Women aged ≥50 years at wave 8.	Women aged ≥45 years in the baseline analytic extract.
Primary analytic sample	4,904 women with complete three-wave loneliness, baseline burden, and primary composite.	2,080 women eligible for paired cross-validation. Complete-case primary association model: 2,025 women.	228,807 eligible women; 182,571 in the complete-case baseline-composite model. Depression: 174,398; grip: 182,228; CRP: 172,134.
Baseline age in primary analytic sample	Mean 56.95 years (SD 8.16); range 45–101.	Mean 64.67 years (SD 8.32); range 50–96, in the 2,080 women.	Mean 57.65 years (SD 6.80); range 45–71, in the 182,571 women. All eligible women: mean 57.90 years (SD 6.79).
Additional male analyses	4,287 men; mean age 58.16 years (SD 8.11), range 45–88. Sex-comparative total: 9,191.	1,372 men; mean age 64.72 years (SD 7.80), range 50–89. Sex-comparative total: 3,452.	None. The baseline comparison was restricted to women.
Loneliness assessment	2011, 2013, 2015; direct CES-D item about the preceding week. High: ≥3 days per week.	Waves 8, 10, 12 (2006, 2010, 2014); indirect three-item scale. High: mean score ≥2 on a 1–3 scale.	Baseline assessment in 2006–2010; joint indicator requiring often feeling lonely and never/almost never being able to confide.
Outcome window	Psychological, cognitive, ADL, and mobility changes: 2011–2018. Grip, walking time, chair stands, and balance: 2011–2015.	Primary changes: wave 8–13 (2006–2016). In the selected sample, depression, ADL, and cognition contribute; grip change is unavailable.	Baseline symptoms, grip, and CRP; no longitudinal outcome or trajectory comparison in this analysis.
Temporally separated secondary analyses	2015 endpoint-free status to 2018 incident outcome; separate 2015–2018 continuous-change composite adjusted for 2015 burden.	Wave 12 endpoint-free ADL to wave 13 incident limitation; separate wave 12–13 continuous-change models.	Not conducted.
Interpretation	Within-cohort descriptive incremental information over a partly overlapping observation period.	Directional comparison and internal paired model evaluation, not external validation of the CHARLS model.	Baseline association with a different social-disconnection indicator; cannot establish temporal replication or benefit of repeat measurement.

Note. SD, standard deviation; CRP, C-reactive protein. Ages and denominators refer to the selected analysis samples, not the entire parent cohorts. HRS complete-case regression and cross-validation denominators differ because predictor imputation was used in cross-validation. The usual UK Biobank recruitment age range was 40–69 years; the retained age variable in the selected extract ranges up to 71 years.

Cohort descriptions: Zhao et al., International Journal of Epidemiology 2014, doi:10.1093/ije/dys203; Sonnega et al., International Journal of Epidemiology 2014, doi:10.1093/ije/dyu067; Sudlow et al., PLoS Medicine 2015, doi:10.1371/journal.pmed.1001779. Tables S28–S30 describe the measures actually used here.

Supplementary Table S28. CHARLS exposure outcome and covariate definitions.

Measure	Assessment and scoring	Timing	Use and interpretation
Loneliness item	CES-D item felt lonely during the preceding week (harmonized r{w}flonel). Responses: 1, less than 1 day; 2, 1–2 days; 3, 3–4 days; 4, 5–7 days. High loneliness = response 3 or 4.	2011, 2013, 2015	Primary exposure. A single item, not a diagnostic scale; internal consistency is not estimable.
Response patterns	Three binary indicators in chronological order: 000 never high; 100 remitted; 001, 010, or 011 incident; 101 or 110 intermittent; 111 persistent.	2011–2015	Never high is the reference. The incident group includes a high response in 2013 followed by a not-high response in 2015.
Assigned cumulative score	(2×L2011 + 2×L2013 + 3×L2015)/2, with L = 1 for high loneliness; range 0–3.5. Equal-weight sensitivity: L2011 + L2013 + L2015. Continuous sensitivity: raw item minus 1 instead of L; strict binary sensitivity: response 4 only.	Measured 2011–2015; final status assigned through 2018	One primary-score unit approximates two assigned high-loneliness years. Within-interval persistence is assumed, not measured.
Depressive symptoms	CES-D-10 score minus the loneliness contribution (raw loneliness response minus 1), yielding CES-D-9, range 0–27. Higher = more symptoms. Worsening = 2018 minus 2011.	2011 and 2018	Primary psychological domain. Removing the loneliness item reduces direct duplication but not shared affective or reporting influences.
Global cognition	Sum of immediate and delayed word recall (0–20), date orientation (0–4), serial sevens (0–5), and figure copying (0–1); range 0–30, with all four components required. Decline = 2011 minus 2018.	2011 and 2018	Primary cognitive domain. Higher raw scores indicate better performance.
ADL limitations	Harmonized four-item count: difficulty bathing, dressing, eating, and getting into/out of bed; range 0–4. Worsening = 2018 minus 2011.	2011 and 2018	Physical-function component and separate domain outcome. This is not the six-item HRS ADL measure.
Mobility difficulties	Seven-item difficulty count: walking 100 m, climbing stairs, rising from a chair, stooping/kneeling/crouching, raising arms, lifting/carrying, and picking up a small coin; range 0–7. Lower-body count uses the first four items, range 0–4. Worsening = 2018 minus 2011.	2011 and 2018	Two physical-function components. Individual items are not entered again as additional components.
Grip strength	Supplied cleaned dominant-hand grip strength in kg (harmonized gripsum source). Decline = 2011 minus 2015.	2011 and 2015	Physical-function component. Baseline sign-reversed grip also contributes to aligned burden. Analyses retain the supplied cleaned values.
Walking test	Average walking-test time in seconds (harmonized wspeed variable despite its name); retained range 1–60 seconds. Worsening = 2015 minus 2011.	2011 and 2015	Physical-function component. It is elapsed time, not speed in metres per second.
Chair stands and balance	Time for five chair stands, retained range 1–120 seconds; worsening = 2015 minus 2011. Balance score 1–4 from side-by-side, semitandem, and full-tandem performance; deterioration = 2011 minus 2015.	2011 and 2015	Two physical-function components. These outcomes end at the final loneliness assessment.
Physical-function domain	Mean of available standardized changes in grip, ADL, seven-item mobility, lower-body mobility, walking time, chair-stand time, and balance; at least two of seven components required.	Mixed 2011–2015 and 2011–2018	Higher = greater functional worsening. Correlated mobility measures may contribute overlapping information.
Primary composite	Equal-weight mean of available psychological, physical-function, and cognitive domain scores; at least two of three domains required. Physical indicators are averaged before averaging across domains.	Observation period through 2018	Component z scores use available observations in the full 25,586-row source dataset before final-sample restriction. The final mean is not rescaled to SD 1.
Aligned baseline burden	Mean of available standardized CES-D-9, sign-reversed global cognition, sign-reversed grip strength, and ADL limitations; at least two of four required.	2011	Primary models and remission–outcome contrast. Cognition, grip, ADL, CES-D-9 and inflammation models use their corresponding baseline measure; walking and chair-stand models have no baseline performance adjustment. Physical-function, mobility and metabolic models use the broader score defined below.
Broader baseline burden	Mean of standardized worse cognition, worse grip, ADL, CES-D-9 and log CRP; at least three of five. Components retain full-source standardization; no CKM stage component.	2011	Adjustment for physical-function, mobility, metabolic and remission-correlate models, and the broader/CRP-exclusion sensitivity models. Not the aligned primary baseline score.
Alternative composites	Broader outcome: mean standardized worsening in cognition, grip, ADL, CES-D-9 and log CRP, at least three of five. CRP-exclusion: remaining four, at least three. Depression-exclusion: cognition, grip, ADL and log CRP, at least three. Functional-cognitive: cognition, grip and ADL, at least two.	Mixed 2011–2015 and 2011–2018	All are CKM-free and remain means of standardized components, not scores rescaled to SD 1. Depression-exclusion and functional-cognitive models adjust for baseline worse cognition, worse grip, ADL and log CRP, requiring at least three of four.

Measure	Assessment and scoring	Timing	Use and interpretation
Complete-component restriction	Restrict to all five observed broader-composite change components (cognition, grip, ADL, CES-D-9 and log CRP), then fit the original primary outcome and aligned baseline model.	Observation interval	The numerical ≥5 threshold was retained after removing CKM; it now requires five of five components. This selects 1,934 women. It is a primary-outcome completeness sensitivity, not a broader-composite outcome model.
Metabolic and inflammatory outcomes	Mean of standardized 2011–2015 changes in HbA1c, glucose, log triglycerides, LDL cholesterol, sign-reversed HDL cholesterol, creatinine, and cystatin C; at least three of seven required. Inflammation = standardized change in log CRP. Total cholesterol is not in the implemented metabolic mean.	2011 and 2015	Exploratory only. These changes overlap the full measured exposure window and cannot establish subsequent biological effects.
Age and education	Age in years, centred at 57 in association models. Original education codes 1–2 = low (below completed primary); 3–5 = middle (traditional schooling, primary, middle); 6–10 = high (high school or above). Missing education is a separate category.	2011	Baseline adjustment; education treated categorically.
Residence BMI smoking and alcohol	Rural residence: 1 rural village, 0 urban community. Measured BMI in kg/m². Ever smoked: 0/1. Any alcohol use in the previous year: 0/1.	2011	Baseline adjustment. Definitions concern ever smoking and recent alcohol use, not necessarily current smoking or hazardous drinking.
Chronic-condition count	Sum of available positive doctor-diagnosed reports for hypertension, diabetes, heart disease, stroke, kidney disease, lung disease, cancer, arthritis, emotional/nervous/psychiatric problems, liver disease, asthma, and memory-related disease.	2011	Range 0–12; all 12 responses are not required. Disease-specific sensitivities replace the count with individual indicators.
Social isolation	Unpartnered status plus no social participation, range 0–2. Partnered corresponds to harmonized marital codes 1 or 3; no social participation corresponds to socwk = 0.	2011	Adjustment variable. Measures limited objective disconnection, not perceived support or the quality of relationships.
Covariate missingness	Association models retain original filled covariates. In the 4,904 women, BMI was filled for 588 (12.0%) at 23.09646 kg/m², and one chronic-condition count at 1. Other listed numeric covariate missingness flags were zero in this sample.	Baseline preparation	For CV, source missingness flags were restored and numeric median/categorical-mode imputation was fitted in training folds. Outcomes and exposure histories were not imputed. Source outcome standardization remained fixed.
Incident functional endpoints	Endpoint-free in 2015 and observed in 2018: ADL 0 to >0; seven-item mobility 0 to >0; lower-body mobility 0 to >0; difficulty walking 100 m 0 to 1; chair-rise difficulty 0 to 1. Eligibility is determined separately for each endpoint.	2015 to 2018	Secondary temporally separated outcomes from the full 5,677-woman trajectory-eligible sample, without requiring the primary composite. Final model n varies.
Other incident endpoints	Depressive symptoms: CES-D-9 <10 in 2015 to ≥10 in 2018. Heart disease/stroke: neither reported in 2015 and either reported in 2018, requiring both 2018 disease fields.	2015 to 2018	CES-D-9 ≥10 is an operational symptom threshold, not a clinical diagnosis. Seven cumulative-exposure tests form one FDR family; 28 trajectory contrasts form another.
Strict continuous change	Mean available standardized worsening in cognition, ADL, and CES-D-9 from 2015 to 2018; at least two of three domains. Additional 2015 burden = mean of standardized worse cognition, worse grip, ADL, CES-D-9 and log CRP; at least three of five. No CKM stage component.	2015 to 2018	Separate sensitivity, not the primary composite. The revised 2015-adjusted cumulative-exposure estimate was small and positive; this does not establish causality.

Note. Source variables are from the Harmonized CHARLS D data and the study's analysis-ready dataset. Higher worsening scores indicate poorer health. Available-component means and source-wide standardization do not define a validated ageing scale. To improve measurement transparency, we reviewed the documentation for secondary-outcome construction. The imported CKM-derived measures lacked the full component rules required for explicit reporting; stage progression and stage-derived cardiovascular outcomes were therefore excluded during revision. CKM contributions were also removed from alternative composites, baseline adjustments and completeness counts; affected analyses and FDR values were recalculated. The primary analysis and the distinct 2015–2018 self-reported heart-disease/stroke endpoint were unchanged. Complete upstream cleaning documentation for the supplied grip values remained unavailable.

CES-D, Center for Epidemiologic Studies Depression Scale; ADL, activities of daily living; BMI, body mass index; CVD, cardiovascular disease; CV, cross-validation; FDR, false discovery rate; LDL, low-density lipoprotein; HDL, high-density lipoprotein. CHARLS study documentation: https://charls.pku.edu.cn/en/ .

Supplementary Table S29. HRS exposure outcome and covariate definitions.

Measure	Assessment and scoring	Timing	Use and comparability
Loneliness	Hughes three-item scale: lack of companionship, feeling left out, and feeling isolated. Imported mean score lblonely3 ranges 1–3 after orienting higher responses towards greater loneliness. High = mean ≥2, equivalent to sum ≥6 when all three items are observed.	Waves 8, 10, 12; 2006, 2010, 2014	Indirect scale about frequency of feelings, rather than the CHARLS direct past-week CES-D item. Psychosocial assessments used alternating subsamples at four-year intervals.
Response patterns and cumulative exposure	000 never high; 100 remitted; 001, 010, 011 incident; 101, 110 intermittent; 111 persistent. Cumulative score = L8 + L10 + L12, range 0–3.	2006–2014	One unit is one additional high-loneliness assessment, not two years of exposure. CHARLS and HRS cumulative coefficients have different units.
Depressive symptoms	Eight-item CES-D symptom count (cesd), range 0–8. Worsening = wave 13 minus wave 8, standardized. The loneliness symptom is retained in this outcome.	2006–2016	Unlike CHARLS CES-D-9, this outcome retains loneliness-related content. Direct item duplication is reduced by the indirect exposure scale but symptom overlap remains.
ADL limitations	Six-activity limitation count (adl6a), range 0–6: dressing, walking across a room, bathing, eating, getting into/out of bed, and using the toilet. Worsening = wave 13 minus wave 8, standardized.	2006–2016	Broader activity set than the four-item CHARLS count. Incident limitation uses wave 12 = 0 and wave 13 >0.
Global cognition	Supplied harmonized global cognitive z score (tcog_z_z), higher = better. It combines available standardized memory, executive-function, and orientation domains. Decline = wave 8 minus wave 13, followed by standardization.	2006–2016	Not simply the supplied 27-point cognitive score. Domain availability and source normalization differ from the raw 0–30 CHARLS total; upstream item-level weights and reference norms are not specified in the retained extract.
Grip strength	Maximum of supplied left- and right-hand grip values in kg; each hand variable records the maximum trial value. Baseline sign-reversed grip contributes to baseline burden where available.	Wave 8 baseline	Wave-13 grip is missing in all selected women and men; grip change does not contribute to the actual primary or strict-change composite.
Primary composite and baseline burden	Programmed follow-up rule averages available standardized depression, ADL, cognition, and grip changes, requiring at least three. In the final sample, exactly depression, ADL, and cognition contribute. Baseline burden averages the corresponding baseline scores, requiring at least three of four.	Wave 8–13 outcome; wave 8 burden	Baseline burden can include grip, whereas the follow-up composite does not. Components are standardized in the source comparison frame; the final mean is not rescaled to SD 1.
Age education and residence	Baseline age in years; supplied harmonized three-level education raeducl (low, middle, high; categorical codes 1–3); binary rural residence.	Wave 8	Core adjustment. Category equivalence to the detailed CHARLS educational system is not assumed.
BMI smoking and alcohol	Self-reported BMI in kg/m²; ever smoked (0/1); drinks alcohol (0/1).	Wave 8	The HRS BMI is self-reported, whereas CHARLS uses measured BMI. Alcohol definitions are not identical across cohorts.
Chronic conditions	Available positive reports of doctor-diagnosed hypertension, diabetes, heart disease, and stroke; at least two of four indicators required to compute the count.	Wave 8	Fewer conditions than the 12-condition CHARLS count. Disease-specific sensitivity replaces the count with these four indicators.
Adjustment and missing data	Association models use outcome-specific complete cases, categorical education, and HC3 standard errors. Models adjust for the corresponding baseline domain or baseline composite; the cognition model uses baseline composite. HRS models do not include the CHARLS social-isolation score.	Baseline covariates	CV: numeric median and categorical-mode imputation plus encoding are fitted within training folds. Exposure history and outcomes are not imputed; this explains larger CV denominators.
Post-exposure outcomes	Wave-12 to wave-13 changes use the same depression, ADL, and cognition measures; separate adjustment includes wave-12 burden. Incident ADL restricts to no limitation at wave 12 and assesses any limitation at wave 13.	2014–2016	Incident-ADL complete-case RR model: 1,731 women, 155 events. The CV incident-ADL sample has 1,873 women, 164 events because predictor imputation differs. These analyses remain secondary.

Note. HRS is a directional longitudinal comparison, not measurement-equivalent external validation. The global cognitive score and harmonized categorical variables were analysed as supplied. Complete upstream item-level scoring and normalization documentation was unavailable.

Scale source: Hughes et al., Research on Aging 2004, doi:10.1177/0164027504268574. Psychosocial questionnaire documentation: Smith et al., HRS Psychosocial and Lifestyle Questionnaire 2006–2022, doi:10.7302/24695. RAND HRS documentation: https://www.rand.org/well-being/social-and-behavioral-policy/centers/aging/dataprod/hrs-data.html .

Supplementary Table S30. UK Biobank baseline exposure outcome and covariate definitions.

Measure	Assessment and scoring	Timing or units	Use and interpretation
Joint loneliness and low-confiding indicator	Loneliness component: yes to often feeling lonely (Field 2020 = 1). Low-confiding component: never or almost never able to confide (Field 2110 = 0). Exposure = 1 only when both are present; otherwise 0. Component sum ranges 0–2, but the model uses the joint binary indicator.	Baseline instance 0	10,014 of 228,807 eligible women (4.38%) meet both criteria. The comparison group includes one-component and neither-component participants. Not equivalent to CHARLS or HRS loneliness measures.
Social isolation	Sum of living alone (Field 709 = 1), visits from friends/family about monthly or less (Field 1031 codes 4–7), and no listed weekly social/leisure activity (Field 6160 = −7); range 0–3.	Baseline instance 0	Mutually adjusted with the joint loneliness indicator. The visits threshold includes monthly contact; it is not restricted to less-than-monthly contact.
Depressive symptom score	Sum of depressed mood frequency (Field 2050) and lack of interest/pleasure frequency (Field 2060), each for the preceding two weeks. Valid item responses 1–4 are recoded to 0–3; sum range 0–6. Both items required; negative response codes treated as missing.	Baseline, score 0–6	Standardized before analysis. Symptom severity, not clinical depression. Distinct from CES-D-9 in CHARLS and CES-D-8 in HRS.
Grip weakness	Negative of the mean of left- and right-hand grip strength in kilograms (Fields 46 and 47), standardized so higher = weaker grip. The retained bilateral summary was used without changing the original missing-value exclusions.	Baseline, kg	A cross-sectional physical phenotype, not decline. All 226,903 observed retained grip values were consistent with the bilateral mean.
Inflammation	Serum C-reactive protein (Field 30710), with values ≤0 set to missing, natural-log transformed, then standardized.	Baseline, mg/L before transformation	Not equivalent to change in CRP. A small baseline association cannot validate or refute the longitudinal CHARLS biomarker findings.
Baseline composite	Mean of available standardized depressive symptoms, grip weakness, and log-CRP; at least two of three components required. Standardization occurs in the merged source dataset before female/age restriction; no final SD-1 rescaling.	Baseline only	Contains inflammation but not cognition or ADL. It is not the CHARLS psychological-functional-cognitive composite.
Age and deprivation	Age in years and Townsend deprivation index as supplied in the baseline dataset; higher Townsend score = greater area deprivation.	Baseline	Adjustment in all displayed baseline models.
Education	Binary indicator of a college/university degree versus no degree; imported education level 1 versus 0, corresponding to qualifications Field 6138.	Baseline	Different from the three-level education covariate in CHARLS and HRS.
BMI and smoking	Measured BMI in kg/m². Smoking: 0 never, 1 previous, 2 current (Field 20116).	Baseline	Smoking is entered numerically in the implemented model, not as a set of category indicators.
Alcohol intake	Imported three-level frequency: 1 never; 2 special occasions through once/twice per week; 3 three or more times per week. Derived from alcohol-frequency Field 1558.	Baseline	Entered numerically as 1–3. These categories are not grams of alcohol and are not harmonized to the binary CHARLS/HRS measures.
Physical activity	UK Biobank-derived summed days activity (Field 22033, instance 0): the sum of days of walking, moderate activity, and vigorous activity; range 0–21. The supplied field was entered numerically without recalculation from individual questions.	Baseline, summed activity-days per week	This is an activity-frequency sum, not MET-minutes/week or a count of unique active calendar days. A calendar day can contribute to more than one activity category.
Statistical model and missingness	OLS with HC3 standard errors. Covariates: age, Townsend deprivation, education, BMI, smoking, alcohol, physical activity, and the other social-disconnection exposure. Outcome-specific complete cases; no imputation in these models.	Baseline only	Outcome/covariate-complete n: composite 182,571; depression 174,398; grip 182,228; log-CRP 172,134. No chronic-disease adjustment or male-specific analysis was performed.
Comparison scope	Analytic extract contains baseline exposures and phenotypes only. UK Biobank itself includes repeat assessments, but repeat loneliness was not used in this analysis.	2006–2010 initial assessment	Supports a baseline comparison in women, not replication of trajectories, prospective risk, or the incremental-information result.

Note. Field numbers refer to UK Biobank Data Showcase baseline instance 0. The combined exposure and social-isolation rules were checked against the retained raw question responses. Negative/missing questionnaire codes were not treated as negative findings in the displayed complete-case models; the retained exposure file already contained valid responses. The activity covariate was verified as Field 22033; supplied values from the original extract were retained rather than recomputed using a later derivation update.

UK Biobank Data Showcase: https://biobank.ndph.ox.ac.uk/showcase/ . Relevant fields: loneliness 2020; confiding 2110; household size 709; visits 1031; weekly activities 6160; symptoms 2050 and 2060; grip 46 and 47; CRP 30710; qualifications 6138; smoking 20116; alcohol 1558; summed days activity 22033. Confiding coding 100501, visits 100327, activities 100328, and symptom frequency 100484 provide the response labels. Activity definition: https://biobank.ndph.ox.ac.uk/showcase/field.cgi?id=22033 .
